# The yeast protein kinase Sch9 adjusts V-ATPase assembly/disassembly to control pH homeostasis and longevity in response to glucose availability

**DOI:** 10.1371/journal.pgen.1006835

**Published:** 2017-06-12

**Authors:** Tobias Wilms, Erwin Swinnen, Elja Eskes, Laura Dolz-Edo, Alice Uwineza, Ruben Van Essche, Joëlle Rosseels, Piotr Zabrocki, Elisabetta Cameroni, Vanessa Franssens, Claudio De Virgilio, Gertien J. Smits, Joris Winderickx

**Affiliations:** 1 Department of Biology, Functional Biology, KU Leuven, Heverlee, Belgium; 2 Department of Molecular Biology and Microbial Food Safety, Swammerdam Institute for Life Sciences, University of Amsterdam, GE Amsterdam, The Netherlands; 3 Department of Biology, University of Fribourg, Fribourg, Switzerland; University of Utah, UNITED STATES

## Abstract

The conserved protein kinase Sch9 is a central player in the nutrient-induced signaling network in yeast, although only few of its direct substrates are known. We now provide evidence that Sch9 controls the vacuolar proton pump (V-ATPase) to maintain cellular pH homeostasis and ageing. A synthetic sick phenotype arises when deletion of *SCH9* is combined with a dysfunctional V-ATPase, and the lack of Sch9 has a significant impact on cytosolic pH (pHc) homeostasis. Sch9 physically interacts with, and influences glucose-dependent assembly/disassembly of the V-ATPase, thereby integrating input from TORC1. Moreover, we show that the role of Sch9 in regulating ageing is tightly connected with V-ATPase activity and vacuolar acidity. As both Sch9 and the V-ATPase are highly conserved in higher eukaryotes, it will be interesting to further clarify their cooperative action on the cellular processes that influence growth and ageing.

## Author summary

The evolutionary conserved TOR complex 1 controls growth in response to the quality and quantity of nutrients such as carbon and amino acids. The protein kinase Sch9 is the main TORC1 effector in yeast. However, only few of its direct targets are known. In this study, we performed a genome-wide screening looking for mutants which require Sch9 function for their survival and growth. In this way, we identified multiple components of the highly conserved vacuolar proton pump (V-ATPase) which mediates the luminal acidification of multiple biosynthetic and endocytic organelles. Besides a genetic interaction, we found Sch9 also physically interacts with the V-ATPase to regulate its assembly state in response to glucose availability and TORC1 activity. Moreover, the interaction with the V-ATPase has consequences for ageing as it allowed Sch9 to control vacuolar pH and thereby trigger either lifespan extension or lifespan shortening. Hence, our results provide insights into the signaling mechanism coupling glucose availability, TORC1 signaling, pH homeostasis and longevity. As both Sch9 and the V-ATPase are highly conserved and implicated in various pathologies, these results offer fertile ground for further research in higher eukaryotes.

## Introduction

In *Saccharomyces cerevisiae*, Sch9 is part of the highly conserved TORC1 pathway which plays a central role in the nutrient-induced signaling network, thereby affecting many aspects of yeast physiology such as stress resistance, longevity and cell growth [1–3]. The rapamycin-sensitive TORC1 mediates these effects mainly via two key branches. In the first branch, activated TORC1 phosphorylates and inhibits Tap42, which in turn controls the activity of type 2A and type 2A-like protein phosphatases [4, 5]. In the second branch, TORC1 contributes to Sch9 activation by phosphorylating multiple residues in its C-terminus [6]. In addition to TORC1-mediated activation, Sch9 can be phosphorylated by the sphingolipid-dependent Pkh1-3 kinases on the conserved PDK1 site, and this phosphorylation is indispensable for its function [6, 7]. Although Sch9 is a downstream effector of the TORC1 complex, the protein kinase can also function independently of TORC1 [3, 8, 9]. Moreover, it has been proposed that Snf1, the orthologue of mammalian AMP kinase, also modulates Sch9 activity by phosphorylation [10]. As three different kinases modulate Sch9 activity in response to diverse stimuli, Sch9 appears to act as a major integrator that regulates various aspects of yeast physiology. A prime example of this is the control of lifespan by Sch9. Indeed, both *tor1Δ* and *sch9Δ* strains display increased lifespan as compared to the WT strain [2, 11], and downregulation of nutrient signaling via the TORC1-Sch9 branch seems to be part of an evolutionary conserved mechanism that extends lifespan across a wide range of eukaryotic species [12, 13].

The V-ATPase is a highly conserved proton pump that mediates the luminal acidification of multiple organelles of the biosynthetic and endocytic pathway, thereby regulating numerous cellular processes including vesicle trafficking, autophagy, pH- and ion- homeostasis (reviewed in [14–16]). These V-ATPases are multi-subunit protein complexes consisting of a membrane-embedded V_0_ sector containing the proton translocation pore, and an attached peripheral cytosolic V_1_ sector responsible for ATP hydrolysis to fuel proton transport. Although higher eukaryotes often exhibit tissue- and/or organelle-specific expression of multiple isoforms of one subunit, in yeast only the V_0_ sector *subunit a* is encoded by organelle specific homologues: *VPH1* encodes the isoform localized at the vacuole, while *STV1* encodes the isoform that cycles between the Golgi apparatus and endosomes [17].

In both yeast and higher eukaryotes, V-ATPase activity is tightly regulated by reversible assembly of the V_1_ and V_0_ sector [14, 18–20]. Although the exact molecular mechanism governing glucose-dependent reversible assembly is still a matter of debate, recent reports shed some light on the signaling mechanisms by which yeast might control this assembly process. Addition of glucose to carbon starved cells triggers an increase in cytosolic pH (pHc), possibly through a rise in ATP levels. The V-ATPase responds to pHc by assembling and transducing the cellular signal through distinct GTPases, ultimately leading to enhanced Ras/PKA and TORC1 activity. As a result, cells adapt growth in response to carbon source availability [21, 22]. Besides fermentable sugars, additional signals, such as osmotic stress and high extracellular pH, also influence V_0_-V_1_ assembly levels [22–24]. Interestingly, increasing evidence suggests that V-ATPase activity is required for regulating cell survival in both yeast and higher eukaryotes [25–29].

To better understand the mechanisms by which Sch9 regulates cell physiology in yeast, we performed a genome-wide synthetic genetic array (SGA) screening and identified mutants that require Sch9 function for their growth and survival. Gene ontology (GO) analysis revealed the V-ATPase as one of the most significant hits. Further analysis showed that Sch9 physically interacts with the V-ATPase, thereby influencing V-ATPase assembly/disassembly in response to glucose availability, while receiving input from TORC1. Importantly, we show that the interaction of Sch9 with the V-ATPase is required to allow proper control of both pHc and vacuolar pH (pHv), and we found that particularly pHv is important to determine longevity.

## Results

### SGA screening points towards a role for Sch9 in vesicular trafficking and V-ATPase function

**Fig 1:**
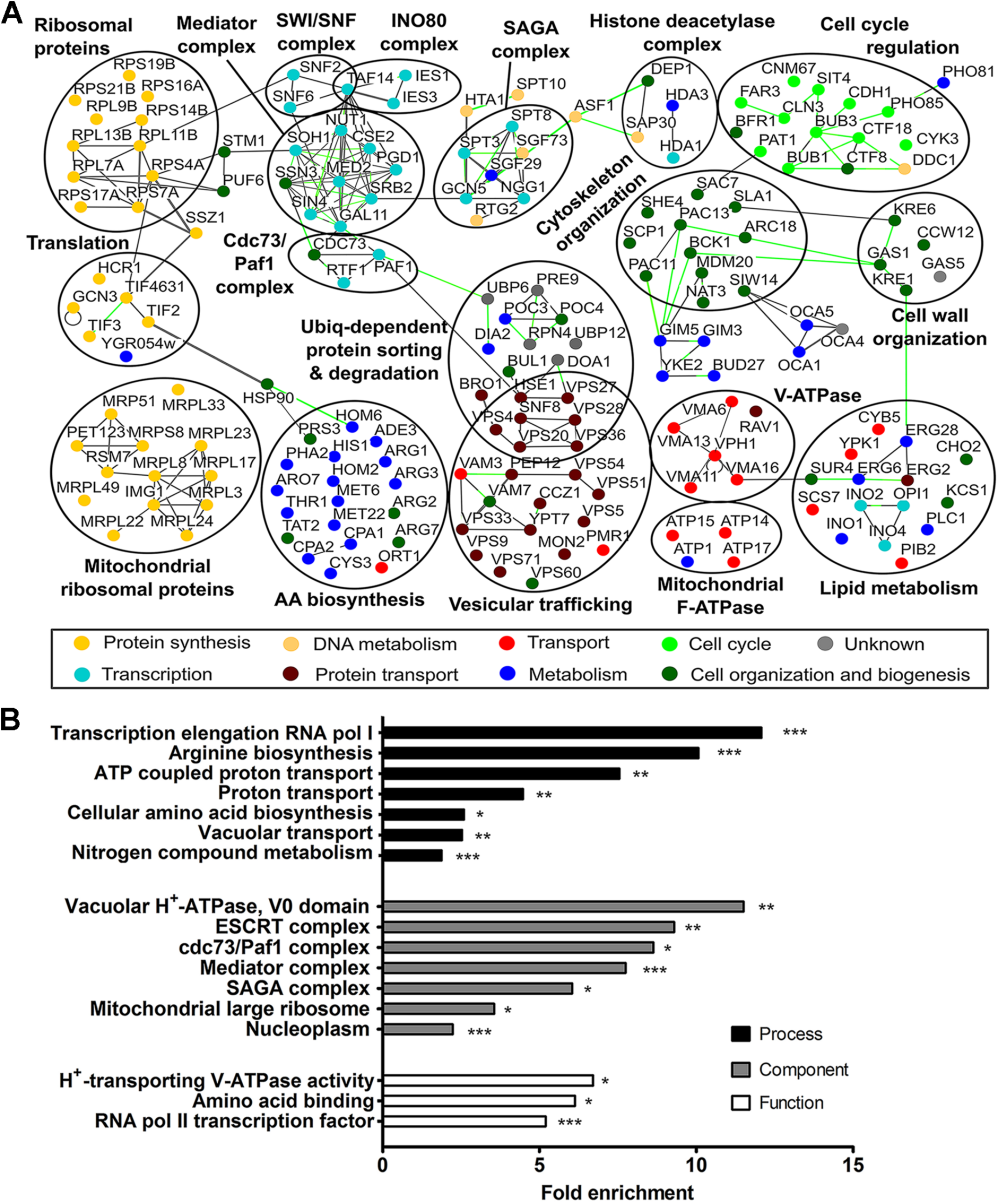
Systematic analysis of genetic interaction partners of *SCH9*. (A) *SCH9* genetic interaction network. Osprey software was used to graphically display the relationships between genes identified in the SGA screening. Synthetic lethal interactions are connected by green lines; protein-protein interactions are shown in gray. Nodes are colored by process. Circles indicate well-defined protein complexes or group of genes that are functionally related. For clarity reasons, not all identified genes, nor all known interactions are shown. (B) Hypergeometric enrichment organized according to GO function, process and component. See also **S1 and S2 Tables**.

As only a few direct substrates are known for Sch9 [30–32], we performed a genome-wide SGA analysis to discover additional functions and targets. To this end, the *sch9Δ* mutant was mated with the library of non-essential deletion strains and double deletion mutants were scored for a synthetic growth phenotype. GO analysis on the identified genes **(S1 Table)** revealed several functional classes for which a role of Sch9 had already been established, such as transcription, protein synthesis, mitochondrial function and cellular amino acid biosynthesis [3, 32, 33], validating the approach. Interestingly, our screening also identified numerous new genes displaying a genetic interaction with *SCH9* as only for a small subset of these genes (± 15%) a putative interaction with *SCH9* has been predicted by BioGRiD **(S1 Table)**. Two GO categories that were significantly enriched in our screening, but not represented in BioGRID are connected to vesicular trafficking and V-ATPase function **(S2 Table and Fig 1)**.

### General protein trafficking is not compromised in the *sch9Δ* mutant

**Fig 2:**
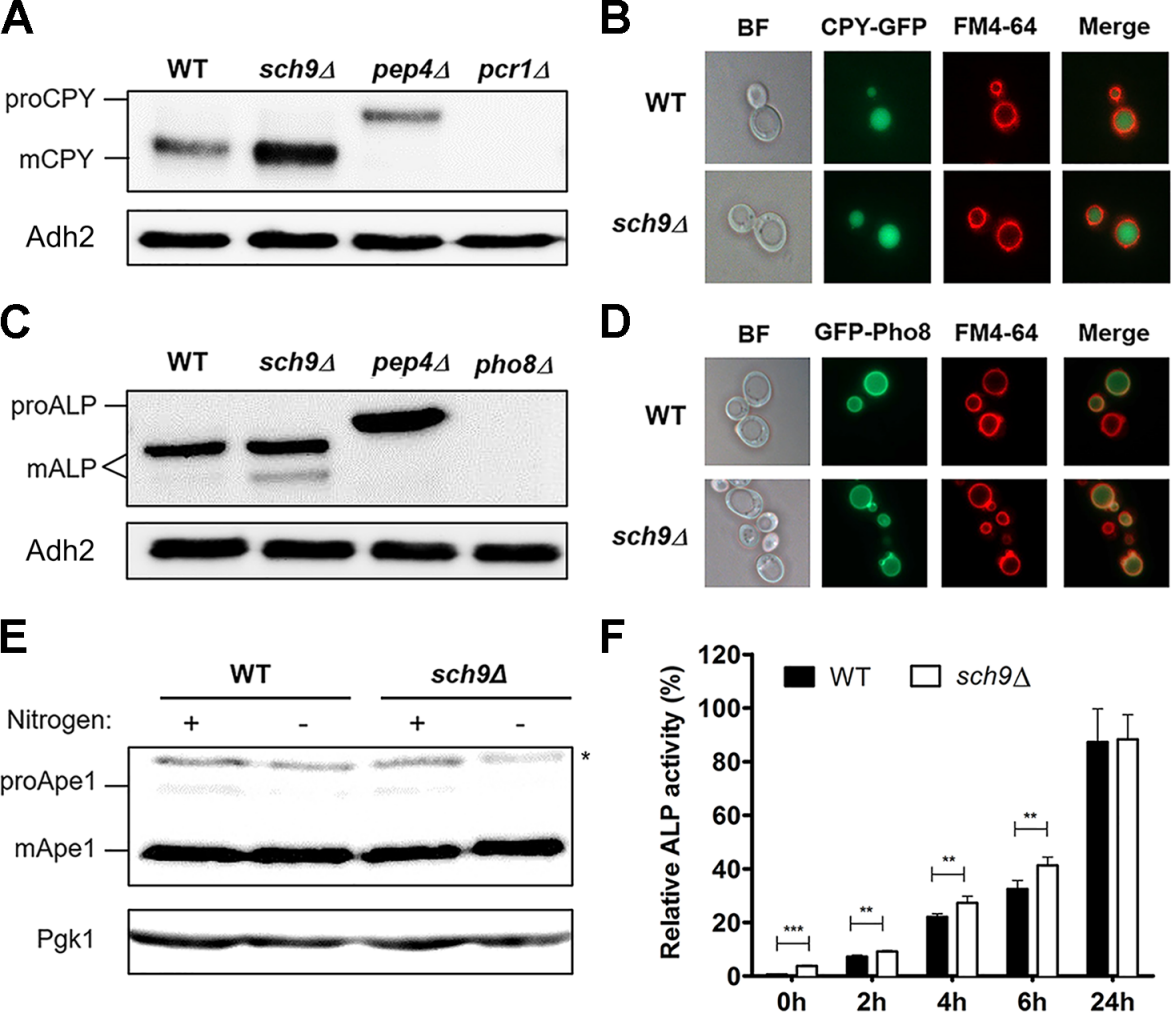
Sch9 does not impact on vesicular trafficking. (A-E) Sorting and processing of vacuolar proteases is not impaired in exponentially growing *sch9Δ* cells. Processing of CPY (A), ALP (C) and Ape1 (E) was examined by Western blot. * represents cross-reacting band. Intracellular localization of CPY-GFP (B) and GFP-Pho8 (D) was examined by fluorescence microscopy. (F) Sch9 affects basal and non-specific autophagy. Exponentially growing cells expressing Pho8*Δ*60 were shifted to nitrogen starvation medium. Samples were taken at the indicated time points, proteins extracted, and specific activities determined. Results are normalized to the activity of a WT strain starved for 24h. Mean values ± SD are shown (unpaired t-test). See also **S1 and S2 Figs**.

We first investigated whether the synthetic interaction between *SCH9* and genes encoding proteins involved in vacuolar protein sorting arises from a defect in vesicular trafficking in the *sch9Δ* mutant. To this end, we monitored the localization of several marker proteins known to be sorted to the plasma membrane or vacuole by one of different trafficking routes **(S1 Fig)** [34]. Fig 2 **(Fig 2A and Fig 2B)** shows that the soluble hydrolase carboxypeptidase Y (CPY) was correctly sorted to the vacuolar lumen in the *sch9Δ* mutant, where it was processed to its mature form. Interestingly, deletion of *SCH9* results in increased abundance of CPY **(Fig 2A and S2A Fig)**, and, in contrast to vacuolar protein sorting mutants [35], CPY was not secreted from the cell in the *sch9Δ* strain **(S2B Fig)**, additionally confirming the CPY pathway is not impaired. The CPY receptor Vps10, which is recycled from the late endosome (LE) to the trans-Golgi network (TGN) after CPY dissociation [36], localizes to punctate endosome and Golgi compartments in both WT and *sch9Δ* mutant cells, suggesting that retrograde transport from LE to TGN is not compromised in the *sch9Δ* strain **(S2C Fig)**. Mutants affecting early endosome function mislocalize the v-SNARE protein Snc1. In agreement with previously data [37], most of the GFP-Snc1 fluorescence was present at the plasma membrane in both WT and *sch9Δ* cells and only a fraction of the protein was observed in internal cellular structures **(S2D Fig)**. This result indicates that the secretion of proteins to the cell surface, as well as the recycling of proteins from the cell surface to the TGN is not impaired in the *sch9Δ* mutant. Additionally, the successful staining of internal membranes of *sch9Δ* cells with FM4-64 indicates that *SCH9* deletion does not impair endocytosis nor impact vacuolar morphology in exponentially growing cells (**Fig 2B and 2D)**.

Proteins are also transported to the vacuole by non-endosomal routes. Indeed, the alkaline phosphatase (ALP) route directly transports proteins from the TGN to the vacuole [38]. Moreover, the selective cytoplasm-to-vacuole-targeting (Cvt) and non-specific autophagy pathways deliver cytosolic proteins to the vacuole [39, 40]. As can be seen in Fig 2 **(Fig 2C and 2E)**, the vacuolar hydrolases ALP, encoded by *PHO8*, and Ape1 are processed to their active form in *sch9Δ* cells, suggesting that Sch9 does not play an essential role in the ALP or Cvt pathway, respectively. In line with this, Sch9 did not influence GFP-Pho8 localization to the vacuolar membrane **(Fig 2D)**. Previous studies have implicated TORC1, Sch9 and PKA in negatively regulating autophagy [8, 41]. We used the Pho8*Δ*60 and GFP-Atg8 processing assays to monitor the delivery and lysis of autophagic bodies in the vacuolar lumen [42]. Although we observed a small increase in basal and starvation induced autophagic flux in the *sch9Δ* mutant **(Fig 2F and S2E Fig)**, this small difference is unlikely to explain the observed synthetic growth defects when combining the deletion of *SCH9* with that of genes encoding proteins involved in vesicular trafficking. Moreover, none of the *ATG* genes were retrieved in our SGA screening. Collectively, our results show that Sch9 does not directly control vesicular trafficking pathways for protein transport to the vacuole, the plasma membrane, or for endocytosis.

### Sch9 impacts on pH homeostasis

**Fig 3:**
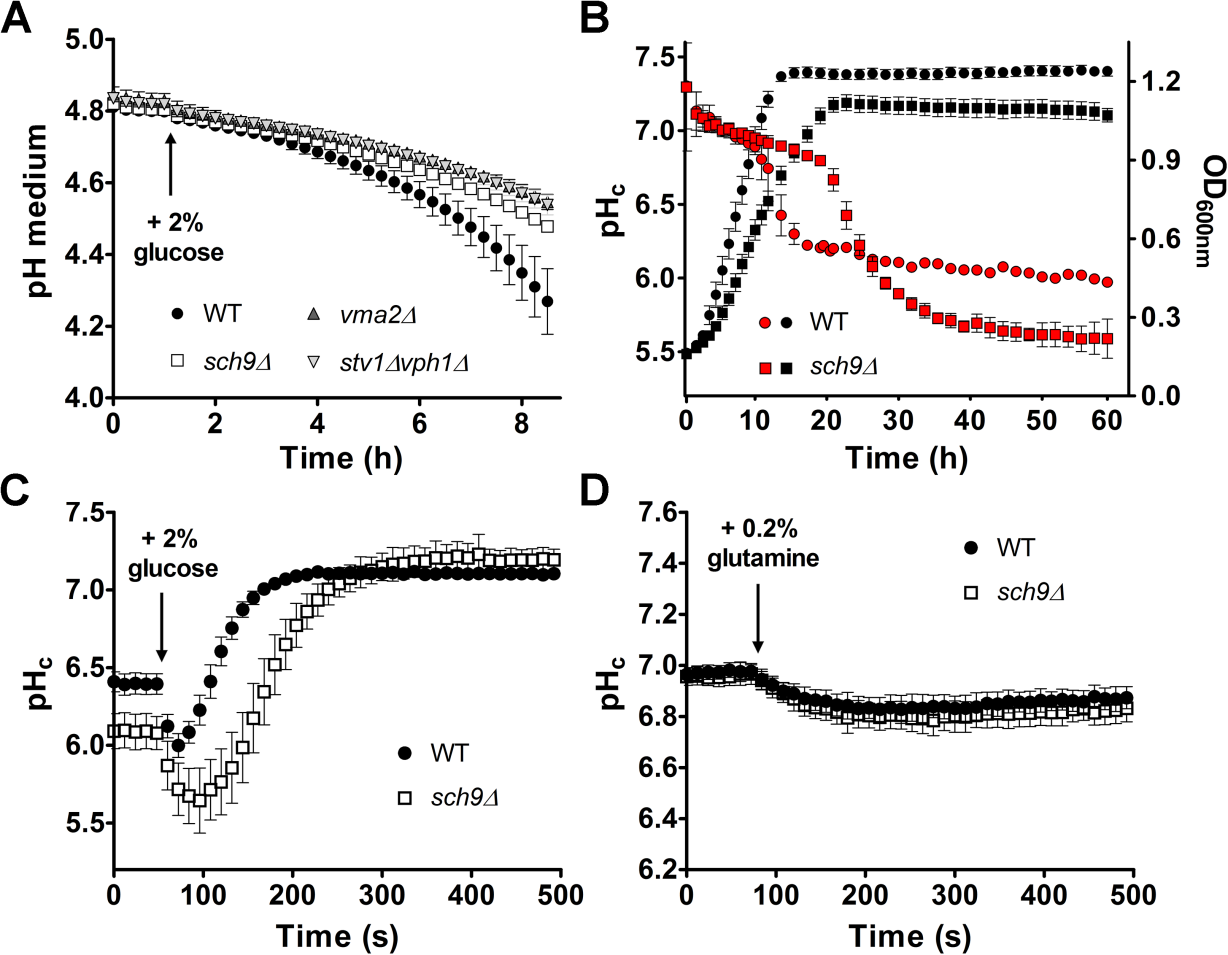
Sch9 influences pH homeostasis. (A) Glucose-induced medium acidification is compromised in the *sch9Δ* mutant. Exponentially growing cells were starved for glucose and proton export was initiated by the addition of 2% glucose. A representative experiment with 3 independent samples for each strain is shown. (B) Sch9 affects pHc upon depletion of glucose. Growth (black) and pHc (red) were simultaneously monitored. A representative experiment with 5 independent samples for each strain is shown. Error bars in (A, B) represent SD from the mean. (C) pHc recovers more slowly in the *sch9Δ* mutant. Glucose-starved cells were pulsed with 2% glucose. (D) Nitrogen depletion does not affect pHc. Nitrogen-starved cells were pulsed with 0.2% glutamine. Data points in (C, D) show the average of at least 14 replicates, error bars represent 95% confidence intervals (CI). See also **S3 Fig**.

Another GO category that was significantly enriched in our screening is connected to the vacuolar proton pump **(Fig 1)**. Since the V-ATPase is a key regulator of pH homeostasis [43] and since the majority of Sch9 localizes to the vacuolar membrane, where also the V-ATPase is found [17, 44], we investigated whether the deletion of *SCH9* affected pH. As a first indication, we measured the ability to acidify the extracellular medium upon re-addition of glucose to glucose-starved cells. Consistent with a potential role in maintaining pH homeostasis, we found that cells lacking *SCH9* displayed a reduced glucose-activated proton export similar as been reported for cells lacking a functional V-ATPase (**Fig 3A**) [43]. Next, we monitored the effects on pHc. We could not observe a difference in pHc between WT and *sch9Δ* cells during fermentative growth **(Fig 3B)**, although *sch9Δ* cells maintained their neutral pHc longer due to slower growth and, consequently, later depletion of glucose. However, after the diauxic shift, the pHc of *sch9Δ* cells dropped below that of WT **(Fig 3B)**. Similarly, a more acidic pHc for the *sch9Δ* mutant was observed when cells were starved for carbon **(Fig 3C and S3A Fig)**. In line with previous data [45], subsequent re-addition of glucose to WT starved cells resulted in a rapid acidification of the cytosol followed by an alkalization to neutral pH within 3 minutes after the glucose pulse. In contrast, the *sch9Δ* mutant displayed a retarded recovery of pHc after glucose re-addition as it reached neutral pH values only 5 minutes after the pulse **(Fig 3C)**. Whereas the rapid acidification step is believed to be caused by initiation of glycolysis, subsequent alkalization of the cytosol is the result of coordinated activation of the plasma membrane H^+^-ATPase Pma1 and the vacuolar V-ATPase. Hence, similar to *sch9Δ* cells, the pHc of mutants with a dysfunctional V-ATPase also recovers more slowly after glucose deprivation [43]. Thus, the data described above are all consistent with a functional link between Sch9 and the V-ATPase.

Because Sch9 is a known effector of the nitrogen-responsive TORC1 complex, we asked whether nitrogen starvation, similarly to glucose starvation, may influence pHc in a Sch9-dependent manner. Interestingly, pHc remained unchanged following nitrogen starvation in both WT and *sch9Δ* cells **(Fig 3D and S3B Fig)**.

### Synthetic sick phenotype upon combining deletion of *SCH9* with mutants which lack all V-ATPase activity

**Fig 4:**
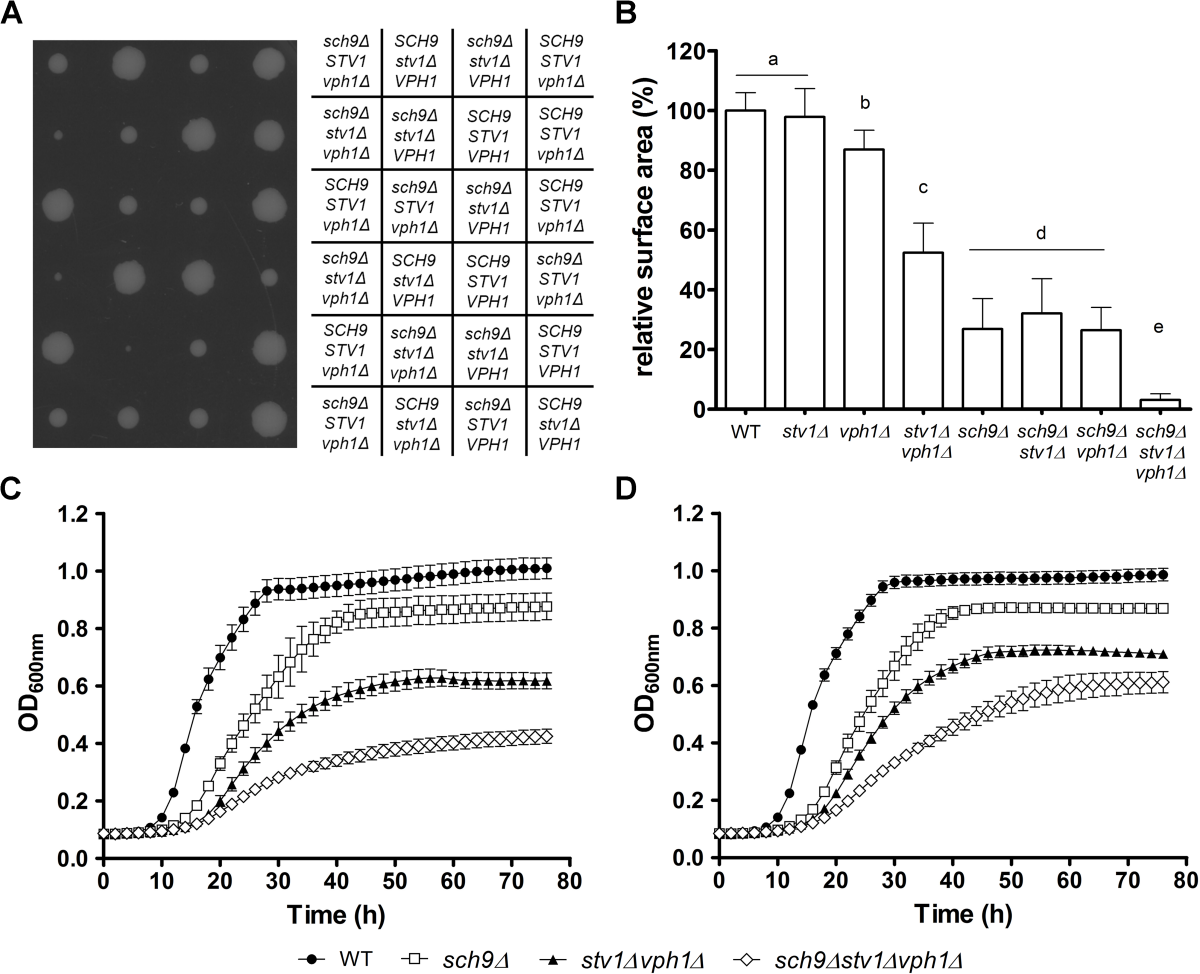
Effects on colony size and growth by deletion of *VPH1*, *STV1* and/or *SCH9*. (A, B) A synthetic sick phenotype arises when deletion of *SCH9* is combined with a fully dysfunctional V-ATPase. (A) Tetrad dissection of the diploid strain JW 04 952 (*sch9Δ/SCH9 vph1Δ/VPH1 stv1Δ/STV1*). (B) Colony sizes were calculated, normalized relative to WT and are shown as mean values ± SD. Letters indicate groups of strains with a significant difference in colony size (p < 0.001, one-way ANOVA). (C, D) Strains combining deletion of *SCH9* with a fully dysfunctional V-ATPase show a deteriorated growth phenotype. OD_600nm_ was followed over time in fully supplemented medium without buffer (C) or buffered at pH 5 (D). A representative experiment with at least 4 independent colonies for each strain is shown. Error bars represent SD from the mean. See also **S4 and S5 Figs**.

Because of the high-throughput nature of our SGA screen, we decided to manually validate the genetic interaction between Sch9 and the V-ATPase using tetrad dissection. Remarkably, all mutants that combined the deletion of *SCH9* with the deletion of a *vma* subunit displayed a synthetic sick phenotype **(Fig 4A and S4 Fig)**. A quantitative measure of the synthetic fitness defect was obtained by measuring colony sizes using Image J **(Fig 4B and S5A Fig)**. Consistent with the genetic interaction, we observed a severe deteriorated growth phenotype of these mutant strains on fully supplemented medium, even when this medium was buffered at pH 5 to fully support growth of the V-ATPase mutants **(Fig 4C and 4D, S5B–S5E Fig)**. Interestingly, our analysis confirmed the partial functional redundancy of the V_0_ subunit *a* isoforms [17], as it required the simultaneous deletion of *VPH1* and *STV1* to observe a synthetic growth phenotype in combination with the deletion of *SCH9* **(Fig 4 and S5 Fig)**. Taken together, we established a negative genetic interaction between *SCH9* and the V-ATPase complex and show that this is a general phenomenon that cannot be attributed to a specific V-ATPase subunit or sector.

### The *sch9Δ* mutant has a partial *vma*^*-*^ phenotype

To gain insight into the molecular mechanisms underlying the genetic interaction, we sought processes that are regulated by both Sch9 and the vacuolar proton pump. To this end, we assayed growth on media known to impair growth of either the *sch9Δ* or V-ATPase deficient mutants [9, 14]. Similar growth defects were observed for both *sch9Δ* and various V-ATPase mutants when they were exposed to osmotic stress or grown on media with high calcium concentrations. In addition, both types of mutants were unable to grow on non-fermentable carbon sources and on YPD medium containing 60 mM CaCl_2_ buffered at pH 7.5 **(Table 1 and S6 Fig)**. Interestingly, as both Sch9 and V-ATPase activity are required for tolerance to rapamycin and manganese, two substances known to affect TORC1 signaling [6, 46], our results point towards a functional relationship between TORC1 and the V-ATPase. Unlike V-ATPase deficient mutants, however, the *sch9Δ* mutant is tolerant to zinc and high extracellular pH, but the additional deletion of *SCH9* does not restore growth of the V-ATPase deficient mutants on these two media **(Table 1)**. On the other hand, the deletion of *SCH9* appears to cause enhanced sensitivity of the *stv1Δ* or *vph1Δ* strains to osmotic stress and elevated extracellular calcium, and to partially impair growth on non-fermentable carbon sources. In general, it seems that phenotypes which rely on the H^+^ pumping ability of the V-ATPase are only mildly influenced by the deletion of *SCH9*, while mostly the phenotypes that are not readily attributable to the vacuole acidifying function of the V-ATPase, *i*.*e*. the non-canonical functions, appear to be affected by loss of Sch9. Taken together, these results show that the *sch9Δ* mutant has a partial *vma*^*-*^ phenotype and suggest that Sch9 may somehow regulate the V-ATPase.

**Table 1:** Phenotypic overlap between *sch9Δ* and mutants in which V-ATPase function is impaired.

Investigated phenotype	WT	*sch9Δ*	*vmaΔ*	*stv1Δ*	*vph1Δ*	*stv1Δ vph1Δ*	*sch9Δ vmaΔ*	*sch9Δ* *stv1Δ*	*sch9Δ vph1Δ*	*sch9Δ* *stv1Δ vph1Δ*
YPD (pH 6.5)	++	++	++	++	++	++	++	++	++	++
YPD, pH 5.0	++	++	++	++	++	++	++	++	++	++
**YPD, pH 7.5**	++	**++**	**- -**	++	++	- -	- -	++	++	- -
YP + 2% Galactose	++	+	+	++	++	+	-	+	+	- -
YP + 3% Glycerol	++	- -	- -	++	++	- -	- -	-	-	- -
YP + 3% Lactate	++	-	- -	++	++	- -	- -	-	-	- -
YP + 0.3% Lactate	++	+	+	++	++	+	+	+	+	- -
YPD + 150 mM CaCl_2_	++	-	- -	++	+	- -	- -	- -	- -	- -
YPD + 60 mM CaCl_2_, pH 7.5	++	-	- -	-	-	- -	- -	- -	- -	- -
YPD + 0.5 mM BAPTA	++	-	-	++	++	-	- -	-	-	-
YPD + 1 M NaCl	++	- -	- -	++	++	- -	- -	- -	- -	- -
YPD + 2 mM MnCl_2_	++	-	- -	-	++	- -	- -	- -	+	- -
**YPD + 4 mM ZnCl**_**2**_	++	**++**	**- -**	++	- -	- -	- -	++	-	- -
YPD + 50 nM Rapamycin	++	-	- -	+	+	- -	- -	+	+	- -

(++) indicates no sensitivity of the tested strain to the investigated phenotype, (+) limited sensitivity, (-) elevated sensitivity, (- -) hypersensitivity (no observable growth). See also **S6 Fig**.

### The role of Sch9 in lifespan determination switches from pro-ageing to pro-survival upon impairment of the V-ATPase

**Fig 5:**
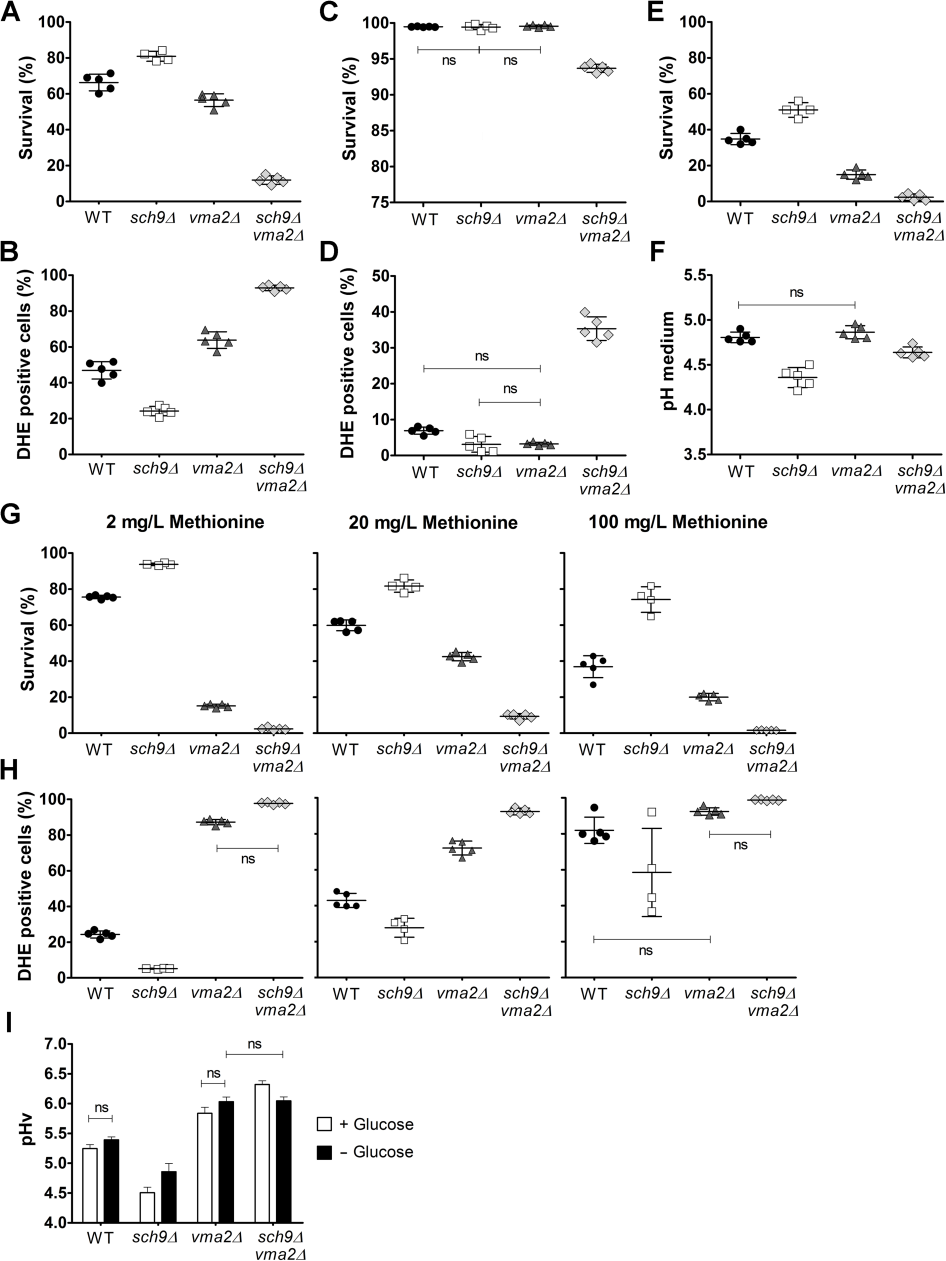
Function of Sch9 in regulating ageing is dependent on V-ATPase activity. With the exception of panel E, all chronological ageing data represent measurements performed on cells at day 8 in stationary phase. (A) Cell survival and (B) ROS determination of strains aged in non-buffered fully supplemented medium as determined by flow cytometry. (C) Cell survival and (D) ROS levels of strains aged in fully supplemented medium buffered at pH 5.5 as determined by flow cytometry. (E) Cell survival of strains grown in buffered medium at day 23 in stationary phase as determined by CFU counting. (F) pH of the culture medium of ageing cells grown in buffered medium. (G) Cell survival and (H) ROS determination of strains grown in medium containing the indicated concentration of methionine as determined by flow cytometry. Results depicted are mean values ± SD. (I) Sch9 affects pHv. Vacuolar pH was measured during exponential growth and during glucose starvation using the ratiometric fluorescent pH indicator BCECF-AM. Results depicted are mean values ± SEM of four independent experiments. All differences between strains and conditions are statistically significant unless stated as ns (not significant). A detailed statistical analysis is presented in **S3 Table**. See also **S7 and S8 Figs**.

One of the best studied phenotypes associated with the loss of Sch9 is the extension of chronological lifespan (CLS) [2, 12, 13]. Although V-ATPase activity has been implicated in regulating yeast ageing [25, 29], not much is known about the effect on CLS when the V-ATPase function is abrogated in *S*. *cerevisiae*. Hence, we determined the CLS of mutants lacking *SCH9* and/or the V-ATPase subunit encoded by *VMA2*. To this end, cells were grown in complete synthetic medium with 2% glucose and viability of cells was measured 8 days after they entered stationary phase. In line with previously published data [2, 11], we observed an increased lifespan for *sch9Δ* cells as compared to WT. In contrast, the *vma2Δ* mutant displayed a significantly reduced viability **(Fig 5A and S7A Fig)**. Interestingly, the CLS decreased dramatically in the *vma2Δsch9Δ* mutant even when compared to *vma2Δ* mutant alone. This suggests that when V-ATPase activity is compromised, Sch9 is important for maintaining cell viability. Very similar results were obtained when we extended our analysis to other V_0_ and V_1_ subunits **(S8A and S8B Fig)**. Thus, the role of Sch9 in lifespan determination is highly dependent on the presence of a functional V-ATPase.

As especially superoxide anions are detrimental to survival and as both Sch9 and V-ATPase activity have been implicated in oxidative stress resistance [11, 26], we assessed the levels of endogenous superoxide anions using dihydroethidium (DHE) during chronological ageing. We found that in stationary phase cells the level of this reactive oxygen species (ROS) was elevated in the *vma2Δ* mutant when compared to WT cells and this difference became more pronounced as cells aged **(Fig 5B and S7B Fig)**. In *sch9Δ* cells, the level of ROS also increased during ageing, but here the amount of ROS was always significantly lower than in WT cells. In agreement with the reduction in CLS, the additional deletion of *SCH9* in the *vma2Δ* mutant resulted in a striking increase in cells showing DHE staining, indicating that superoxide-induced oxidative stress may represent one of the main factors contributing to the rapid ageing of this mutant. Indeed, only a minor fraction of the *vma2Δsch9Δ* cells stained with DHE did not accumulate SYTOXgreen at day 8 in stationary phase **(S8C Fig)**. Moreover, the fraction of cells displaying SYTOXgreen staining but no accumulation of superoxide anions was negligible in all investigated strains (< 0.2%).

It has been shown that extracellular acidification is an important extrinsic factor affecting CLS [47–50]. Hence, we also determined CLS of the WT and mutant strains in complete synthetic medium buffered to pH 5.5. As shown, buffering of the medium indeed reduced mortality significantly as the WT and single *sch9Δ* and *vma2Δ* strains maintained their viability during the first week of chronological ageing, while there was only a small drop in survival for the *vma2Δsch9Δ* mutant (**Fig 5C and S7C Fig**). Again, an inverse correlation was seen with the ROS levels measured under the same conditions (**Fig 5D and S7D Fig**). However, when cells were allowed to age for a longer period, it was once more evident that also in buffered medium Sch9 promoted ageing in case of a functional V-ATPase, while it supported survival when V-ATPase function was compromised (**Fig 5E**). Of note, we also measured the pH of the buffered medium in the aged cultures. We noticed enhanced acidification of the culture medium when cells were lacking Sch9, while the medium pH of aged WT and *vma2Δ* cells was higher and similar. Thus, the pH of the medium cannot explain why the role of Sch9 in regulating longevity switched from pro-ageing to pro-survival upon impairment of the V-ATPase (**Fig 5F)**.

The amino acid composition of the growth medium can affect yeast CLS [51, 52], with methionine availability having a highly significant impact [53]. Indeed, several studies demonstrated that genetic or dietary restriction of methionine promotes longevity [54, 55]. Because our strains are all in the BY4741 background and thus contain a deletion of *MET15*, and since we retrieved *MET6* and *MET22* from the genome-wide SGA screening (**Fig 1 and S1 Table**), we wondered whether methionine availability would differentially influence the CLS of our strains. To this end, strains were aged in non-buffered synthetic medium containing different concentrations of methionine. For WT and *sch9Δ* mutant cells, lifespan decreased with increasing methionine supply, while for cells with a dysfunctional V-ATPase, *i*.*e*. the *vma2Δ* and *vma2Δsch9Δ* cells, survival was better in medium containing the standard 20 mg/L methionine than in medium containing lower or higher concentrations of the amino acid **(Fig 5G and S7E Fig)**. These data confirm that CLS depends on methionine availability and is determined in part by V-ATPase function [55]. Nonetheless, independent of methionine availability, the deletion of *SCH9* still extended longevity, while it reduced longevity when combined with disruption of the V-ATPase activity. Again, a tight correlation between survival and superoxide levels could be observed for all methionine concentrations tested **(Fig 5H and S7F Fig)**.

Since our data indicate that Sch9 impacts on pH homeostasis and since several studies have linked yeast ageing to V-ATPase activity and vacuolar acidification [25, 29, 56], we also measured pHv in WT and mutant strains to determine whether this could explain the apparent differential roles of Sch9 in regulating CLS. We used the pH-sensitive fluorescent dye BCECF-AM and performed measurements in cells growing exponentially on glucose, as well as in glucose-starved cells. As compared to WT cells, the sole deletion of *SCH9* was associated with a significant drop in pHv in both conditions, indicative for enhanced V-ATPase activity **(Fig 5I)**. Remarkably, when the *SCH9* deletion was introduced in the strain lacking Vma2, it caused an increase in pHv. This effect was only apparent in cells growing on glucose, suggesting that especially under these conditions Sch9 controls pHv also independently of the V-ATPase. Because the changes in pHv correlated well with the CLS profiles for the strains studied, our data are in line with a model in which vacuolar acidity dictates cellular longevity.

### Sch9 physically interacts with the V-ATPase and influences its assembly

**Fig 6:**
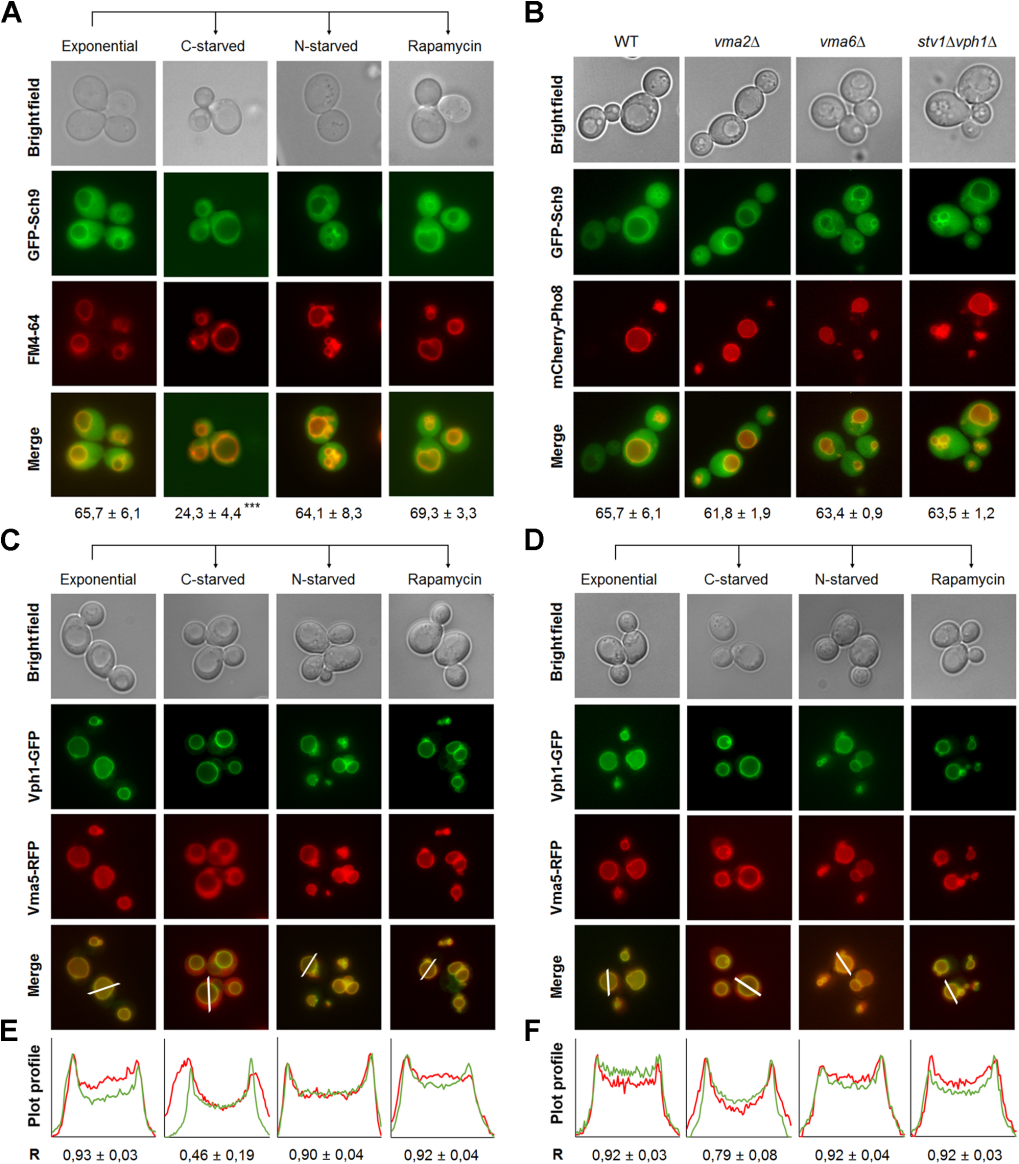
Dynamic localization of Sch9 and its regulation of V-ATPase disassembly. (A) Vacuolar membrane enrichment of Sch9 is regulated by glucose availability. Cells expressing GFP-Sch9 were grown to exponential phase in minimal medium buffered at pH 5 and stained with FM4-64. Next, cells were washed in starvation medium and deprived of either glucose or nitrogen for 30 min. Alternatively, cells were treated with 200 nM rapamycin. For each condition, vacuolar membrane localization of GFP-Sch9 was assessed for at least 750 cells from three to four independent experiments. Mean values ± SD are shown. A one-way ANOVA analysis was performed to designate statistical differences. (B) V-ATPase activity does not mediate Sch9 localization. Cells of the indicated genotype co-expressing GFP-Sch9 and mCherry-Pho8 were grown to exponential phase in minimal medium buffered at pH 5 and their intracellular localization was analyzed by fluorescence microscopy. Vacuolar membrane localization of GFP-Sch9 was assessed in at least 600 cells from two to three independent experiments. Mean values ± SD are shown. (C-F) Sch9 regulates V-ATPase disassembly in response to glucose availability. WT (C) and *sch9Δ* (D) cells co-expressing Vma5-RFP and Vph1-GFP were grown as in **Fig 6A** and their intracellular localization was analyzed by fluorescence microscopy. (E, F) Combined fluorescence intensity profile plots of Vma5-RFP (red) and Vph1-GFP (green) measured along the line displayed in the merged panel for WT (E) and *sch9Δ* (F) cells. The x-axis depicts the distance along the line in pixels, while the y-axis indicates the relative RFP or GFP signal intensities. The Pearson’s coefficient (R) ± SD was calculated using the ImageJ plugin JACoP. See also **S9 Fig**.

Both Vph1-containing V-ATPase complexes and Sch9 are known to locate at the vacuolar membrane during fermentative growth. Hence, we reasoned they might physically interact. Prior to studying this interaction, we identified conditions that influenced the intracellular localization of Sch9 or the assembly state of the V-ATPase. In agreement with previous work [33], GFP-Sch9 was found to be enriched at the vacuolar membrane in exponentially growing cells, but a significant portion dissociated from the vacuolar membrane upon glucose starvation **(Fig 6A)**. In contrast, the protein kinase remained stably associated with the vacuolar membrane upon nitrogen starvation and rapamycin treatment **(Fig 6A)**, as well as in V-ATPase deficient mutants **(Fig 6B)**, indicating that the intracellular localization of Sch9 is specifically regulated by C-source availability. Concerning V-ATPase assembly, we found that the V-ATPase was fully assembled in both WT and *sch9Δ* cells during exponential growth, as well as upon nitrogen starvation and rapamycin treatment **(Fig 6C and 6E, S9A Fig)**. However, when *sch9Δ* cells were subjected to glucose starvation a significant fraction of Vma5-RFP remained localized with Vph1-GFP at the vacuolar membrane, as indicated by fluorescence intensity profile plots (**Fig 6D and 6F, S9B Fig)** and by the Pearson’s coefficient (R) **(S9C Fig)**. Thus, the disassembly of the V-ATPase is apparently hampered in *sch9Δ* cells, which may explain why the cells have a lower pHv as described above.

**Fig 7:**
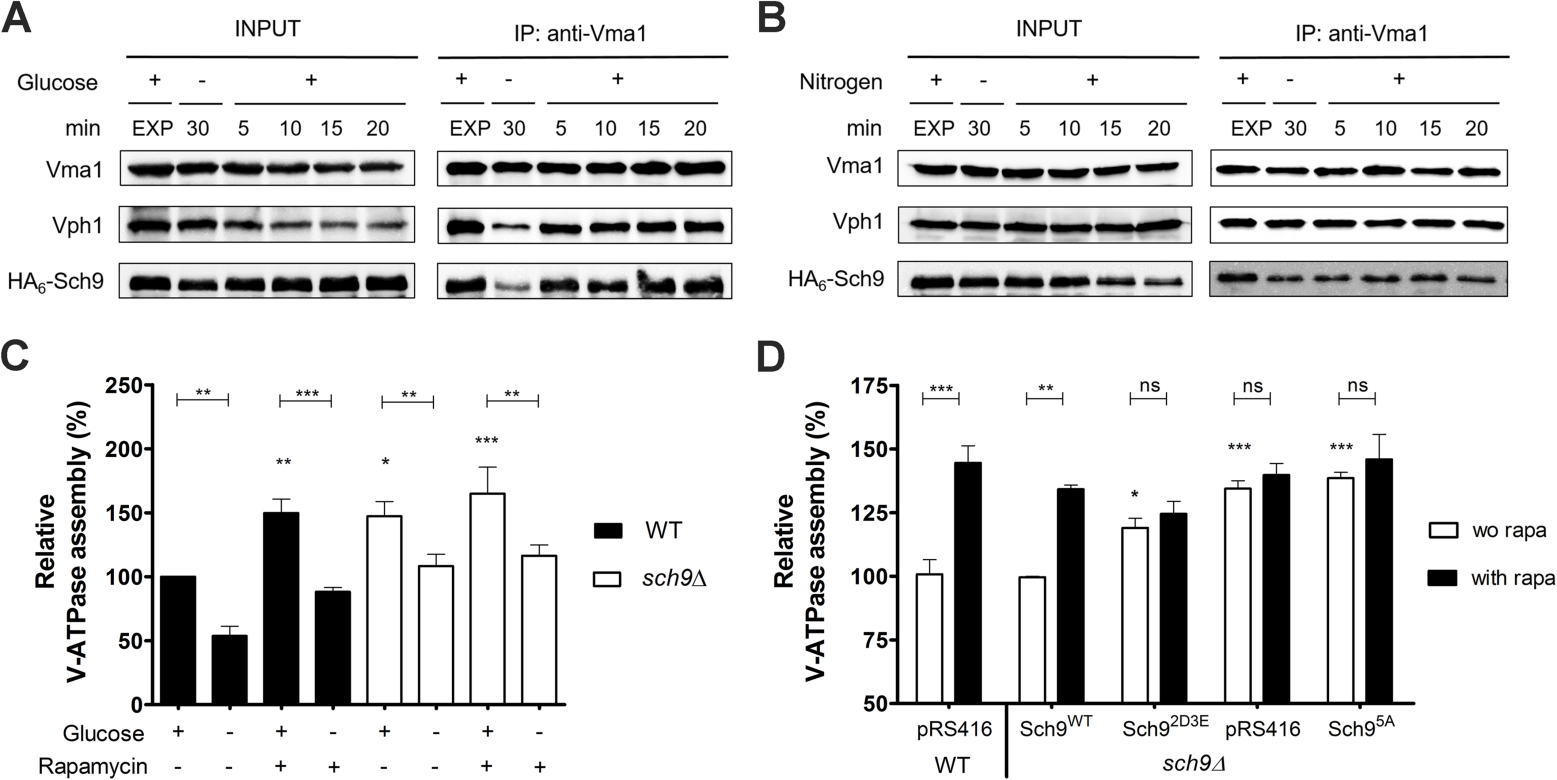
Sch9 physically interacts with the V-ATPase. (A, B) Physical interaction of Sch9 with Vma1 depends on glucose availability. Cells expressing HA_6_-Sch9 were grown as in **Fig 6A**, followed by re-addition of 2% glucose (A) or 0.2% glutamine (B). Total lysates (input) and anti-Vma1 immunoprecipitates (IP) were analyzed by immunoblotting. (C, D) Sch9 regulates V-ATPase assembly downstream of TORC1. (C) WT and *sch9Δ* cells were grown to mid-log phase in YPD, pH 5. Half of the culture was treated with 200 nM rapamycin for 30 min and subsequently starved for glucose in the presence of rapamycin. The untreated half was further grown for 30 min and subsequently starved for glucose. (D) V-ATPase assembly was assessed in the *sch9Δ* strain expressing the empty vector (pRS416), the wild-type *SCH9* gene (Sch9^WT^), or one of the *SCH9* mutant genes in which its TORC1 phosphorylation sites are mutated (Sch9^5A^ and Sch9^2D3E^). The WT strain expressing the empty vector was taken as an additional control. Precultures were grown overnight in minimal medium lacking uracil buffered at pH 5 and inoculated in YPD medium (50 mM MES, pH 5). Once cells reached exponential phase, half of the culture was treated with 200 nM rapamycin (rapa) for 30 min. To quantify V-ATPase assembly, complexes were IPed with antibodies against Vma1 and Vph1. Results are depicted as mean values ± SEM from at least three independent experiments. One- or two-way ANOVA analyses were performed to determine statistical significances. Unless indicated otherwise, asterisks indicate a statistical significance compared to the WT strain grown in YPD without rapamycin. See also **Table 2**.

Since microscopic analyses did not allow quantifying an absolute value of assembled V-ATPase complexes, we further studied the V-ATPase assembly state and the putative interaction of Sch9 with the V-ATPase by co-immunoprecipitation (co-IP). Accordingly, **Fig 7A** shows that in exponentially growing cells a strong interaction between the V_1_ and V_0_ sectors, and between the V-ATPase and HA_6_-Sch9 could be observed. Upon glucose depletion, both interactions weakened considerably, but were rapidly restored by re-supplementation of glucose. In contrast, nitrogen deprivation did not impact on either interaction **(Fig 7B)**; at most there was a slight decrease in the interaction between Vma1 and Sch9. As this interaction was not strengthened by the subsequent supplementation of nitrogen, the minor decrease cannot be attributed to a starvation effect. Concerning V-ATPase assembly levels in WT and *sch9Δ* cells, the results in **Fig 7C** indicate that in exponentially growing cells, both the deletion of *SCH9*, or the treatment of WT cells with rapamycin, significantly increases V-ATPase assembly as compared to untreated WT cells. Because the effect of rapamycin is comparable to that triggered by the deletion of *SCH9*, the data suggest that Sch9 functions downstream of TORC1 to modulate V-ATPase assembly **(Table 2)**. In line with our microscopy data, the absence of Sch9 also significantly lowered the amount of V-ATPase that disassembled upon glucose-starvation. This effect is only partially mimicked when WT cells are treated with rapamycin, suggesting that Sch9 may facilitate glucose starvation-induced V-ATPase disassembly to some extent independently of TORC1. Importantly, glucose depletion still triggered V-ATPase disassembly in the absence of the Sch9, indicative that the role of this kinase is only modulatory.

**Table 2:** Effects of *sch9Δ* and rapamycin on V-ATPase assembly and disassembly.

Condition	Fold change	Adjusted P-value
**Glucose-induced fold decrease** ^a^		
WT (YPD *vs* YP)	1.91	0.0063
*sch9Δ* (YPD *vs* YP)	1.39	0.0072
**Rapamycin effect on glucose-induced fold decrease** ^a^		
WT (YPD + R *vs* YP + R)	1.70	0.0001
*sch9Δ* (YPD + R *vs* YP + R)	1.40	0.0017
**Rapamycin-induced fold increase** ^a^		
WT (YPD *vs* YPD + R)	1.50	0.0016
*sch9Δ* (YPD *vs* YPD + R)	1.10	ns
**Sch9-induced fold increase** ^**b**^		
WT (YPD) vs *sch9Δ* (YPD)	1.47	0.0122
WT (YPD + R) vs *sch9Δ* (YPD)	1.02	ns
WT (YPD + R) vs *sch9Δ* (YPD + R)	0.94	ns

^a^ Statistical significance was determined using a paired one-way ANOVA analysis (Holm-Sidak's multiple comparisons test).

^b^ Statistical significance was determined using an unpaired one-way ANOVA analysis (Holm-Sidak's multiple comparisons test).

To confirm our data, we repeated the co-IP experiments using strains expressing different Sch9 mutants. First, we monitored the effect of glucose availability on V-ATPase assembly in a strain expressing the analog-sensitive *sch9*^*as*^ allele [33], the activity of which can be blocked using the ATP-analog 1-NM-PP1. As compared to our previous data with WT cells, even without inhibitor more V-ATPase remained assembled in the *sch9*^*as*^ strain upon glucose-starvation, but consistent with the data obtained for the *sch9Δ* mutant, more V-ATPase assembly was observed when Sch9 activity was blocked by 1-NM-PP1, and this independently of whether the cells were exponentially growing or glucose starved (**S9D Fig**). Next, we tested the requirement of TORC1-dependent Sch9 phosphorylation by comparing rapamycin-induced V-ATPase assembly in WT and *sch9Δ* mutant strains complemented with either wild-type Sch9, the Sch9^5A^ or the phosphomimetic Sch9^2D3E^ alleles [6]. As expected, expression of the wild-type Sch9 allele in the *sch9Δ* mutant restored the V-ATPase assembly state to WT levels and rendered it again sensitive to rapamycin **(Fig 7D)**. In contrast, rapamycin did not significantly affect V-ATPase assembly upon expression of the Sch9 phospho-mutants. While maximal assembly was obtained in the presence of Sch9^5A^, similar as seen for the empty vector control, an intermediate level was found in case of the Sch9^2D3E^ allele. The latter indicates that this TORC1-independent Sch9^2D3E^ allele may not be fully functional in downstream signaling as noted before [57]. Nonetheless, when combined, our results suggest that Sch9 integrates input from TORC1 to influence the glucose-dependent assembly state of the V-ATPase.

## Discussion

### Sch9 integrates nutrient signaling with pHc homeostasis, V-ATPase assembly and growth

By conducting a genome-wide SGA screening, we defined a global *SCH9* genetic interaction network in yeast and, as such, identified numerous new genes that may function as Sch9 effector or act in pathways connected to Sch9. Among these hits were several genes involved in modulating vacuolar biogenesis and function. However, we could not find evidence that Sch9 is involved in regulating trafficking routes that deliver material for vacuolar biogenesis or degradation. Instead, we found this protein kinase to influence pHc, pHv and extracellular acidification. We also demonstrated that Sch9 interacts with V-ATPase subunits to modulate the assembly state of the latter in function of nutrient availability, thereby integrating input from TORC1 and a yet undefined glucose-dependent sensor.

Our findings are consistent with observations made in a recent study that coupled vacuolar biogenesis and functioning to cell growth and cell cycle progression [58]. This study demonstrated that Vph1 and components of the TORC1 complex are delivered to the vacuolar membrane early during vacuole biogenesis, while Sch9 is only recruited at a later stage. Moreover, this study reported that Sch9, along with TORC1, signals the cell-cycle machinery that a functional vacuole is present. Interestingly, Sch9 is thereby not only activated by TORC1-dependent phosphorylation, but also by additional signals that require a functional vacuole [58]. That Sch9 is involved in determining cell growth and division was already known for some time, but it remained mainly connected to a dynamic network that couples nutrient availability with ribosome biosynthesis [33, 59].

Consistent with its prominent role in nutrient storage, the vacuole has emerged as central player regulating nutrient signaling pathways in both yeast and mammals [22, 60], though only few studies have begun to unravel the underlying molecular basis. For instance, Young et al. (2010) conducted a genome-wide screening to identify inositol auxotrophy mutants. The authors concluded that the drop in pHc triggered by glucose starvation releases the transcriptional repressor Opi1 from a lipid-sensor complex in the ER, which then translocates to the nucleus to repress the Ino2/4 transcription factors and as such many phospholipid metabolic genes [61]. Another genome-wide screening examined pHc and cell division rate during fermentative growth [62]. This screening retrieved several mutants that could be classified in different categories depending on their pHc-growth rate relationship. Both screenings not only identified various players and potential sensors involved in pHc signaling, but also provided additional links with Sch9. Indeed, more than 20% of the genes retrieved by each screening overlapped with our SGA screening **(S10 Fig and S1 Table)**. As such, Sch9 seems to emerge as a key player connecting inositol and lipid metabolism with pHc, V-ATPase and growth. Whether this connection relates to the sphingolipid-dependent function of Sch9 [7, 63] or the PI(3,5)P_2_-dependent activation of the V-ATPase [24] and vacuolar recruitment of Sch9 [64] needs to be investigated in more detail, but most likely additional mechanisms are at play. We also compared the data from our SGA screening with those obtained by the Cardenas group who performed a genetic screening for synthetic interactions with *TOR1* [65]. Albeit the latter allowed to link Tor1 signaling with vacuolar functions, the overlap between both screenings was mainly restricted to genes encoding cytoplasmic and mitochondrial ribosomal proteins **(S1 Table)**. This may indicate that Sch9 does signal also independently of TORC1, as suggested before [3, 8, 9].

### Lifespan determination by Sch9 depends on the V-ATPase and vacuolar acidification

One well-established phenotype of the *sch9Δ* strain is its increased survival during stationary phase [2, 12], which is partly due to increased respiration and expression of mitochondrial oxidative phosphorylation subunits [11, 66]. Consistently, CLS extension by deletion of *SCH9* can be blocked and even reversed to lifespan shortening by the additional deletion of respiratory genes, by introducing the *SCH9* deletion in rho^0^ strains that lack functional mitochondria or by pregrowing cells under a different nutritional regime [66–68]. We now report that Sch9 can either extend or shorten CLS depending on the presence of a functional V-ATPase and the vacuolar acidity. This is in line with a previous study that connected CLS to the V-ATPase and autophagy-dependent vacuolar acidification under conditions of methionine restriction [55, 56]. Another study demonstrated that CLS extension by methionine restriction requires activation of the retrograde response pathway to regulate nuclear gene expression in function of mitochondrial activity [54]. Interestingly, our SGA-screening confirmed a genetic link between *SCH9* with *RTG2*, encoding a sensor for mitochondrial dysfunction, *RTG3*, encoding a key mediator of retrograde signaling, and with the methionine metabolism genes *MET6* and *MET22*. Hence, the question arises whether both vacuolar acidification and mitochondrial functioning are part of the same regulatory scenario determining longevity. At least for the control of replicative lifespan this seems to be the case. Here, vacuolar acidification is required to maintain proper pH-dependent vacuolar amino acid storage and this prevents age-induced mitochondrial dysfunction [25]. Furthermore, a systematic gene deletion analysis confirmed that several vacuolar mutants, including V-ATPase mutants, affect mitochondrial functions and display similar phenotypes as mitochondrial petite mutants [69].

Given the previously published link between Sch9 and respiratory capacity [11, 66] and the data presented in this paper connecting Sch9 with the V-ATPase and vacuolar pH, it is well possible that Sch9 is part of a system that monitors vacuolar and mitochondrial function in order to sustain growth and lifespan. Such monitoring system may be quite complex as evidenced by a study that demonstrated additive effects of methionine, glutamic acid and glucose availability on yeast longevity [53]. Interestingly this study also attributed a role to Sch9 in the sensing of methionine and glucose, while implicating Gcn2, a conserved protein kinase that links amino acid sensing with global protein synthesis, in the sensing of glutamic acid [53]. As Vam6/Vps39 regulates the formation of the vacuolar-mitochondrial contact sites involved in lipid transfer [70, 71], and contributes to the activation of TORC1 [44], it could play an important regulatory role in this monitoring system.

### Sch9 as regulator of PKA and TORC1

**Fig 8:**
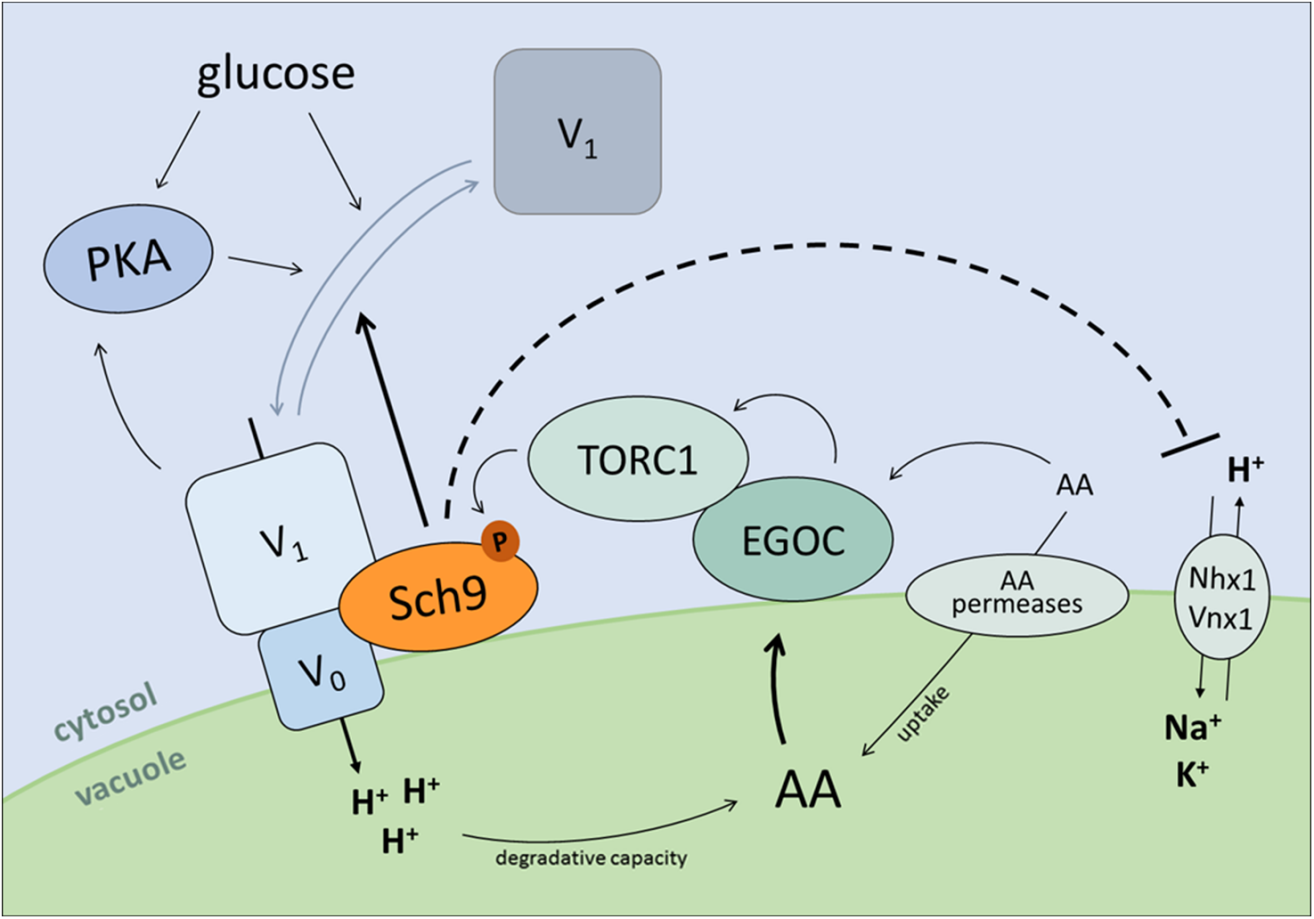
Hypothetical model depicting feedback regulation between Sch9 and the V-ATPase. Sch9 physically interacts with the V-ATPase to modulate its assembly state, thereby affecting vacuolar pH homeostasis. Moreover, Sch9 might also control pHv independently of the V-ATPase by affecting vacuolar proton exchangers. As protein hydrolysis and amino acid uptake are regulated by vacuolar acidity, a feedback mechanism onto Sch9 activity is provided through amino acid sensing by the EGO complex (EGOC) and subsequent regulation of TORC1 activity. Additionally, through modulation of V-ATPase (dis)assembly Sch9 has the ability to indirectly impact on PKA activity. For more details, see text.

In contrast to Sch9, the localization of Tor1 in yeast is not regulated by glucose availability [72]. However, it has been shown that the essential TORC1 subunit Kog1 is transiently sequestered in cytoplasmic stress granules upon heat stress [73] and in cytoplasmic foci in a Snf1-dependent manner upon glucose starvation [72]. As such, the movement of Kog1 in and out of these so-called Kog1-bodies determines the formation of TORC1. Once reconstituted at the vacuolar membrane, the activity of TORC1 is controlled via Vps-C complexes and the amino acid sensing EGO complex (EGOC), the yeast functional counterpart of mammalian Rag-Ragulator [74–76]. Besides being a downstream effector of TORC1, a role of Sch9 in the control of EGOC has not been reported to our knowledge. However, such a role can be suspected given the data we now present on the contribution of Sch9 in regulating V-ATPase assembly and thereby vacuolar acidity. The latter is important for the vacuolar degradative capacity and the pH-dependent storage of amino acids in the vacuolar lumen [25]. In this context, it has been suggested that the Rag GTPases of EGOC could, in addition to mediating cytoplasmic amino acid signals [77], also sense the vacuolar amino acid load through amino acid transport across the vacuolar membrane [74–76]. In addition, and similar to the mammalian system, the Rag GTPase Gtr1 was found to interact with the V-ATPase raising the possibility that the proton pump itself could be involved in the activation of the GTPase [22, 60]. As depicted in **Fig 8**, the consequence of the above is that Sch9 can be part of a feedback loop that keeps V-ATPase and TORC1 activity in balance during growth. The idea of an Sch9-dependent feedback control of TORC1 is not new, because it was already proposed in a previous study that analyzed the effectors by which TORC1 controls the transcription of ribosomal protein and ribosome biogenesis genes [57]. Moreover, both TOR complexes have already been proposed to function in feedback loops to maintain cellular homeostasis [78].

Besides connections with TORC1, the V-ATPase was also identified as signaling intermediate linking C-source availability and pHc with activation of PKA via the GTPase Arf1 [21, 22]. Thus, by regulating V-ATPase assembly, Sch9 would also be upstream of the Ras/PKA pathway. It is known for some time that Sch9 is implicated in the Ras/PKA pathway [79], but only recently it was reported that Sch9 indirectly regulates the phosphorylation of the PKA regulatory subunit Bcy1 via the MAP kinase Mpk1 in a TORC1 dependent manner [80]. Whether this means that Arf1 signals through Mpk1 to control PKA activity needs to be investigated. Importantly, it is known that enhanced PKA activity prevents glucose-induced disassembly of the V-ATPase [81] and therefore also this signaling route is most likely subjected to feedback control.

### Sch9-mediated control of V-ATPase assembly

Several mechanisms have been proposed to control V-ATPase assembly [15, 82]. Of these, the Vph1-specific interactor RAVE (Regulator of ATPase of Vacuoles and Endosomes) might be a good candidate to mediate Sch9 control on V-ATPase assembly, especially since our SGA screening retrieved the gene encoding the central RAVE component Rav1 [83]. However, another possible scenario comes from the observation that during exponential growth on glucose the deletion of *SCH9* was associated with enhanced vacuolar acidification, but when the disruption of *SCH9* was introduced in the strain lacking an active V-ATPase vacuolar alkalization was observed. The reason for this is not known, but it may indicate that Sch9 affects proton exchange by vacuolar antiporters like the Na^+^/H^+^ and K^+^/H^+^ exchangers Nhx1 and Vnx1, both known to play a role in pH homeostasis [84, 85]. If Sch9 would control such antiporters, it would provide a mechanism by which the kinase regulates V-ATPase assembly in response to glucose. Indeed, pH-dependent alterations in the N-terminal cytoplasmic domain of Vph1 has been proposed as possible mechanism governing glucose-dependent reversible assembly of the V-ATPase [86]. This model would explain the observed synthetic genetic interaction between *SCH9* and the genes encoding the V-ATPase. Moreover, it is also in line with the observation that mammalian PKB co-localizes with, phosphorylates and inhibits the cardiac sarcolemmal Na^+^/H^+^ exchanger Nhe1 following intracellular acidosis [87]. This raises again the question whether Sch9 is the genuine orthologue of the mammalian orthologue of PKB/Akt [88, 89] or whether it combines this role with an S6K function in yeast [6].

## Materials and methods

### Yeast strains, plasmids and cell growth

*S*. *cerevisiae* strains and plasmids are listed in **S4** and **S5 Tables,** respectively. All strains used in this study are derived from the BY4741 series. Cells were grown at 30°C in YPD (1% yeast extract, 2% bactopeptone, 2% glucose) or synthetic defined medium (Formedium; 0.5% ammonium sulfate, 0.17% yeast nitrogen base, amino acids, 2% glucose). When indicated, the culture medium was buffered to the specified pH with either MES, sodium citrate or MOPS buffer. For nitrogen starvation experiments, cells were made prototrophic by introducing auxotrophy complementing plasmid(s). For short-term nutrient deprivation, exponentially growing cells were washed with and further grown in starvation medium. For re-stimulation, cells were supplemented with a final concentration of 2% glucose or 0.2% glutamine. For serial dilution growth assays, stationary phase cells were diluted to an OD_600nm_ of 1 and 10-fold serial dilutions were spotted. For growth curve analysis, cultures were grown for 48h and diluted to the same density. OD_600nm_ was measured every two hours in a Multiscan GO Microplate Spectrophotometer (Thermo Scientific).

### Sporulation and tetrad analysis

Diploids were generated by crossing the *sch9Δ* mutant (JW 04 039) with single deletion strains derived from the BY4741 Yeast Knock-out Collection (EUROSCARF) and incubated at room temperature on solid sporulation medium (1% potassium acetate, 1.5% ager) for 5–6 days. A small amount of sporulated cells was resuspended in water containing 0.02 mg/ml lyticase and incubated for 10–15 min at room temperature. Next, tetrads were dissected on a YPD plate using a micromanipulator (Singer instruments). After 3–5 at 30°C, the genotypes of the germinated spores were analyzed based on the segregation of the genetic markers, and/or by PCR analysis. Only deletion mutants with a BY4741 genotype (MATa *his3Δ1 leu2Δ0 met15Δ0 ura3Δ0*) were used in subsequent experiments.

### Synthetic genetic array screening

The *S*. *cerevisiae* deletion strain collection, constructed in the BY4741 background, was obtained from EUROSCARF (Frankfurt, Germany). SGA screening was performed as described previously [90], using the *sch9*::*NATMX4* strain as bait. Colony sizes of single and double mutants were scored by visual inspection. Double mutant strains that displayed an aggravated colony size as compared to the respective single deletion strain and the single *sch9Δ* mutant were retained as potential candidates for a synthetic genetic interaction with *SCH9*.

### Measuring colony size and co-localization with ImageJ

Several interesting candidate genes involved in protein sorting and vacuolar function were manually confirmed using tetrad dissection (see above). After three to four days at 30°C, colony sizes resulting from individual spores were measured using ImageJ software (NIH). Briefly, average colony surface areas were determined using the particle analyzer function for at least seven independent colonies for each genotype. Mutants were categorized as having a negative genetic interaction with *SCH9* when the relative colony size of the double mutant was less than the product of the relative colony sizes of the corresponding single mutants. To quantify the extent of co-localization, the Pearson correlation coefficient was generated using the ImageJ plugin JACoP. Quantifications were performed on different independent experiments, with at least 30 cells analyzed in total. Fluorescence intensity profile plots were created using the Plot Profile function of ImageJ.

### Co-immunoprecipitation

Precultures grown overnight in YPD or minimal medium lacking uracil (Sch9 mutants) were inoculated in fresh YPD medium buffered at pH 5 with 50 mM MES and grown till exponential phase. Next, half of the culture was treated with 200 nM rapamycin (Sigma-Aldrich) for 30 min and subsequently starved for glucose in the presence of rapamycin. The untreated half was further grown for 30 min and subsequently starved for glucose. For V-ATPase assembly analysis by *sch9*^*as*^ inhibition, precultures were diluted in YPD medium (pH 5, 50 mM MES) with or without 300 nm 1-NM-PP1 (Merck-Millipore) and grown for 6 hours, after which cultures were starved for glucose in the absence or presence of the inhibitor. After treatment and/or starvation, cells were collected by centrifugation at 4°C, washed with ice-cold PBS, snap frozen in liquid nitrogen and stored at -80°C. Protein extraction was performed by bead beating in buffer A (40 mM Hepes-NaOH [pH 7.5], 120 mM NaCl, 1 mM EDTA, 0.3% CHAPS, 50 mM NaF, 10 mM β-glycerophosphate) supplemented with complete protease inhibitor tablets without EDTA (Roche). Extracts were cleared by centrifugation and incubated overnight at 4°C with anti-Vma1 or anti-Vph1 (Abcam, 8B1 and 10D7). Next, samples were incubated with magnetic anti-IgG beads (Invitrogen) for 30 min and beads were washed four times with buffer A. Proteins were eluted by boiling in SDS-sample buffer and subjected to immunoblotting. Relative V-ATPase assembly was quantified by calculating the ratio of Vph1 IPed with anti-Vma1 (assembled V_0_V_1_ complexes) *vs* Vph1 levels IPed with both antibodies (free V_0_ and assembled V_0_V_1_) [20].

### Chronological lifespan analysis and determination of ROS levels

Cells were pregrown to stationary phase in non-buffered fully supplemented medium containing 2% glucose. Next, stationary phase cells were diluted to an OD_600nm_ of 0.1 in fresh medium and grown for 48h, which was set as time zero. For standard CLS experiments, cells were aged in non-buffered fully supplemented medium (~ pH 5.5) containing 2% glucose. Buffered CLS experiments were performed in fully supplemented medium buffered at pH 5.5 with 100 mM MES. For CLS experiments with varying methionine concentrations, methionine was added at the indicated concentration to minimal medium lacking methionine. Of note, a standard concentration of 20 mg/L methionine was used throughout all other experiments. At various time points, cell death was measured by flow cytometry (Guava easyCyte 8HT, Merck Millipore) using SYTOXgreen or propidium iodide (Molecular Probes). Alternatively, cell survival was measured by clonogenictiy. To this end, the amount of cells/μl was determined by flow cytometry and 250 cells were plated on YPD agar plates. Subsequently colony forming units were counted and values are displayed as percentage of viable cells. ROS levels were measured via DHE staining and subsequent flow cytometry analysis. Collected flow cytometry data were processed and quantified with FlowJo software.

### Cytosolic, vacuolar and extracellular pH measurements

Cytosolic pH was measured in prototrophic yeast cells expressing pHluorin [45] grown in low fluorescence medium (loflo; Formedium) containing 2% glucose, buffered at pH 5 with 25 mM sodium citrate. Fluorescence emission was recorded at 510 nm using a FLUOstar OPTIMA microplate reader (BMG labtech) with excitation at 390 nm and 470 nm. For starvation experiments, log phase cells were washed twice with starvation medium buffered at pH 5 and fluorescence was measured every 5 min at 30°C for 1h. For pulse experiments, 2% glucose or 0.2% glutamine was administered to starved cells. For growth curve analysis, stationary phase cultures were re-inoculated at the same density in fresh loflo medium and monitored every hour for pHc and OD_600nm_. Calibration was performed by incubating digitonin permeabilized cells in citric acid/Na_2_HPO_4_ buffers of different pH values.

Vacuolar pH was measured as described previously with minor modifications [43]. Briefly, yeast cells were grown to log phase in fully supplemented loflo medium containing 2% glucose, buffered at pH 5 with 50 mM MES and labelled with 50 μM BCECF-AM (Thermo Scientific). Next, cells were washed twice in growth medium with or without 2% glucose and fluorescence was recorded for 30 min at 30°C using a Fluoroskan Ascent FL Microplate Fluorometer and Luminometer (Thermo Scientific). Fluorescence emission was recorded at 538 nm after excitation at 440 nm and 485 nm. Calibration curves were constructed for each strain in every experiment using the calibration mixture described by Brett *et al*. [84], except that the ionophores (monensin and nigericin) were omitted.

The acidification of the culture medium was monitored using the pH indicator bromocresol green sodium salt (BCG; Sigma-Aldrich). For glucose-induced acidification of the medium, cells were grown to exponentially phase in fully supplemented medium containing 2% glucose, buffered at pH 5. Next, cells were washed with glucose starvation medium and resuspended at an OD_600nm_ of 0.1 in starvation medium containing 0.01% BCG. The absorbance of the medium (595 nm) was monitored for 1h in a Multiscan GO Microplate Spectrophotometer. Medium acidification was initiated by the addition of 2% glucose and changes in absorbance over time were recorded. For the measurement of growth media pH of ageing cultures, cells were pelleted and BCG was added to the supernatants at a final concentration of 0.01%. A calibration curve was used to convert the measured absorbance to pH values.

### Autophagy assays and Ape1 processing

Autophagy was monitored using the Pho8Δ60 and GFP-Atg8 processing assay as described previously [91]. Concerning the Pho8Δ60 assay, the generation of p-nitrophenol from p-nitrophenyl phosphate (Sigma-Aldrich, N9389)] was monitored in *pho8Δ* and *pho8Δsch9Δ* mutant strains harboring the *PHO8Δ60* gene by measuring absorbance at 405 nm using a Beckman DTX880 plate reader (Molecular Devices). Specific activities were calculated as nmol p-nitrophenol/min/mg protein. Data are the mean of at least four independent transformants. Concerning the GFP-Atg8 assay, TCA protein extracts were prepared from WT and *sch9Δ* strains harboring the GFP-Atg8-expressing plasmid and equal amounts of proteins were resolved on a 10% SDS-PAGE. Blots were probed with anti-GFP (Roche Diagnostics).

The cytoplasm-to-vacuole pathway was monitored using the prApe1 processing assay as described previously [39]. Briefly, WT and *sch9Δ* cells were harvested, proteins precipitated using the TCA method and equal amounts were loaded on a 10% SDS-PAGE. After western blotting, membranes were probed with anti-Ape1 (kindly provided by Dr. Klionsky).

### CPY secretion

Missorting of CPY was measured by a colony overlay assay as described previously [35]. Briefly, cells were grown to stationary phase and spotted on fully supplemented medium at the indicated OD_600nm_. Plates were placed at 30°C for 4-6h and overlaid with a nitrocellulose membrane. After ± 24h of growth at 30°C, the membrane was washed several times with distilled H_2_O and TBS buffer containing 0.1% Tween-20, and subjected to immunoblotting with anti-CPY (Molecular probes, 10A5).

### Fluorescence microscopy

V-ATPase assembly was investigated by co-localization of pRS315-Vph1-GFP (gift from Robert C. Piper) with Vma5-RFP. To this end, we constructed WT and *sch9Δ* strains expressing a chromosomally encoded, RFP-tagged version of Vma5 by crossing *sch9*::*NATMX4* with RD157 [21]. Correct localization of Sch9 in V-ATPase deficient mutants was examined by co-transforming cells with pRS415-GFP-Sch9 and pRS316-mCherry-Pho8 [92]. Plasmids expressing fusion proteins that served as marker for vesicular compartments were generous gifts (S2 Table). All images were generated using a Leica DM 4000B fluorescence microscope (Leica Microsystems) equipped with a Leica DFC 300G camera.

### Protein extraction, western blot and antibodies

Protein extraction and western blot analysis were performed as described previously [63]. Protein concentrations were determined using the Bradford method (Bio-Rad) or the Pierce 660 nm protein assay (Thermo Scientific). Equal amounts of protein were mixed with SDS-sample buffer and resolved on a SDS-PAGE gel. Either anti-ADH2 (Merck Millipore, AB15002) or anti-PGK1 (Molecular probes, 22C5) were used as loading controls. The ECL method was used for detection and blots were visualized using a UVP Biospectrum Multispectral Imaging System. Signals were quantified by densitometry using UVP VisionWorks LS software (VWR).

### Statistical analysis

Unless stated otherwise, the results shown are mean values and standard deviations displayed as error bars. For other experiments, representative results are shown. The appropriate statistical tests were performed using GraphPad Prism. Significances: * p < 0.05, ** p < 0.01, *** p < 0.001.

## Supporting information

S1 Fig Overview of the investigated protein sorting pathways, as well as their marker proteins. Proteins destined for the vacuole and plasma membrane are sorted in vesicles at the level of the trans-Golgi network (TGN). Upon arrival in the vacuole, proteins are either degraded or cleaved to its active form. The soluble protease proCPY binds the Vps10 receptor in the TGN and this receptor-ligand complex travels to the late endosome. In this compartment, proCPY dissociates from Vps10 and continues its journey to the vacuolar lumen, whereas Vps10p returns to the Golgi via retrograde transport that requires the retromer complex. A direct pathway from the TGN to the vacuole is taken by the vacuolar membrane protein ALP, encoded by *PHO8*. The v-SNARE protein Snc1, which is involved in the fusion of secretory vesicles with the cell surface, takes a route from the TGN directly to the plasma membrane. After vesicle fusion, Snc1 is recycled from the cell surface to the TGN via the early endosome. Endocytosis sorts proteins from the plasma membrane to the vacuole. The lipophilic dye FM4-64 fluoresces strongly after binding to the plasma membrane and is endocytosed to the vacuole by passing through both early and late endosomes. Cytosolic proteins can be transported to the vacuole by either a selective or a non-selective mechanism. The vacuolar aminopeptidase Ape1 is transported into the vacuolar lumen via the selective cytoplasm-to-vacuole-targeting (Cvt) pathway, while GFP-Atg8 and Pho8*Δ*60 are non-specifically transported via autophagy. Both processes use double-membrane vesicles to sequester their cargo. Related to **Fig 2**.
(TIF)

S2 Fig Influence of Sch9 on vesicular trafficking. (A) Sch9 regulates CPY abundance. CPY signals were quantified and normalized for Adh2 levels. Results are expressed relative to the WT strain, which was set at 100%. The mean values ± SD from three independent cultures are shown (unpaired t-test). (B) WT and *sch9Δ* strains do not secrete the soluble hydrolase CPY. Stationary phase cells were spotted and overlaid with a nitrocellulose membrane. After 24h, the membrane was removed, washed and subjected to immunoblotting. (C, D) Sch9 does not influence steady state localization of Vps10-GFP (C) or GFP-Snc1 (D). FM4-64 served as marker for the vacuolar membrane. (E) Delivery and lysis of autophagic bodies is not impaired in the *sch9Δ* mutant. WT and *sch9Δ* strains expressing GFP-Atg8 were grown to exponential phase and shifted to nitrogen starvation medium. At the indicated time points, samples were taken. TCA-extracted proteins were analyzed by immunoblotting using anti-GFP antibody. Related to **Fig 2**.
(TIF)

S3 Fig Effect of Sch9 and nitrogen on pHc. Sch9 affects glucose starvation-induced acidification of the cytosol (A), while nitrogen starvation in general does not impact on pHc homeostasis (B). Cells expressing the pH-sensitive GFP-derivative pHluorin were grown to exponential phase in loflo medium buffered at pH 5, washed twice with starvation medium and transferred to a 96-well microtiter plate. Fluorescence was measured every 5 min for 1h at 30°C in glucose (A) or nitrogen (B) starvation medium. Related to **Fig 3**.
(TIF)

S4 Fig Synthetic sick phenotype of *SCH9* with genes encoding V-ATPase. Diploids, generated by crossing the *sch9Δ* strain (JW 04 039) with the respective single BY4741 deletion strains (EUROSCARF Yeast Knockout Collection), were sporulated and tetrads dissected on YPD (in horizontal rows). Genotypes were determined and are indicated on the right. Related to **Fig 4**.
(TIF)

S5 Fig Genetic interaction of *SCH9* with genes encoding V-ATPase subunits. (A) Examples of quantitative analysis of synthetic sick phenotype. Colony sizes (CS) were calculated with ImageJ, using a minimum of 7 independent colonies for each genotype. CS of single and double deletion strains were normalized relative to WT and the expected colony sizes (ECS) for the double deletion mutants were calculated. Results are shown as mean values ± SD. Letters indicate groups of strains with significant difference in colony size (p < 0.01, one-way ANOVA). (B-E) Growth profiles of the indicated single and double deletion mutants. Growth analysis of *vma2Δ* and *vma6Δ* (B-C), or the semi-redundant V_0_ subunits *stv1Δ* and *vph1Δ* (D-E) of the V-ATPase reveals a growth defect for strains in which a deletion of *SCH9* is combined with a fully dysfunctional V-ATPase. Cultures were pregrown to stationary phase and diluted at the same density in fully supplemented synthetic medium unbuffered (B, D) or buffered to pH 5 (C, E). The mean values ± SD of four independent colonies for each strain are shown. Related to **Fig 4**.
(TIF)

S6 Fig Spot assays of wild type and mutant strains. The *sch9Δ* strain produces a partial *vma*^*-*^ phenotype. Stationary phase cells were diluted to an OD_600nm_ of 1 in growth medium, 10-fold serial diluted and spotted on media known to impair growth of either the *sch9Δ* strain or V-ATPase deficient mutants. (A) Carbon source dependent growth. Various carbon sources were added at the indicated concentration to YP medium. (B) Salt, metal and calcium dependent growth. YPD medium was supplemented with the indicated amount of salt, metal, calcium or calcium chelator. (C) pH sensitive growth. The pH of YPD medium was buffered to pH 5 with 50 mM MES or 7.5 with 100 mM MOPS. (D) Drug sensitivity. Rapamycin was added to YPD medium at a final concentration of 50 nM. Related to **Table 1**.
(TIF)

S7 Fig Flow cytometry analysis of ageing WT and mutant cells over time. (A) Chronological ageing and (B) ROS accumulation over time of strains grown in non-buffered fully supplemented medium. (C) Cell survival and (D) ROS levels of strains grown in fully supplemented medium buffered at pH 5.5 with 100 mM MES. (E) Cell survival and (F) ROS levels of strains grown in medium containing the indicated concentration of methionine. For all experiments, stationary phase cells were inoculated in fresh medium at OD_600nm_ 0.1, grown for 48h (day 0), and stained with SYTOXgreen and DHE at the indicated time points. Results depicted are mean values ± SD. Related to **Fig 5**.
(TIF)

S8 Fig Flow cytometry analysis of WT and mutant cells at day 8 in stationary phase. Chronological ageing of V_1_ (A) and V_0_ subunits (B) of the V-ATPase in combination with deletion of *SCH9* was assessed by staining stationary phase cells with propidium iodide (PI). For each strain, the amount of PI positive (death cells) and PI negative cells (viable cells) was determined. (C) Stationary phase cells were co-stained with SYTOXgreen and DHE to detect loss of membrane integrity and superoxide accumulation, respectively. Results are shown as the average of at least three independent clones for each strain, error bars represent SD. Asterisks indicate a statistical significance compared to the WT strain (one-way ANOVA analysis). Related to **Fig 5**.
(TIF)

S9 Fig Microscopy and co-IP analysis of the V-ATPase assembly state. (A) WT and *sch9Δ* cells co-expressing Vma5-RFP and Vph1-GFP were grown as in **Fig 6A** and their intracellular localization was analyzed by fluorescence microscopy. In contrast to WT, in *sch9Δ* cells a significant portion of Vma5-RFP was still found at the vacuolar membrane during glucose starvation. (B) Fluorescence intensity profile plots. Combined fluorescence intensity profile plots of Vma5-RFP (red) and Vph1-GFP (green) measured along the line displayed in the panels on the left for WT and *sch9Δ* cells. The x-axis depicts the distance along the line in pixels, while the y-axis indicates the relative RFP or GFP signal intensities. (C) The Pearson’s coefficient was calculated using the ImageJ plugin JACoP. Results depicted are mean values ± 95% CI. (one-way ANOVA analysis). (D) V-ATPase assembly and disassembly levels in the *sch9*^*as*^ strain. Cultures were grown in YPD medium with or without 300 nm 1-NM-PP1 for 6 hours, after which they were starved for glucose (30 min) in the absence or presence of the inhibitor. V-ATPase assembly levels were calculated, normalized relative to cells grown on YPD medium without inhibitor and are shown as mean values ± SEM. Statistical significance was tested by a two-way ANOVA analysis (Holm-Sidak's multiple comparisons test). Related to **Figs 6 and 7**.
(TIF)

S10 Fig Venn diagram showing overlap between different genome-wide screenings. A significant overlap of the indicated screenings with our SGA screening implicates Sch9 as central player connecting inositol and lipid metabolism with nutrient availability, pHc, V-ATPase activity and growth.
(TIF)

S1 Table Genes genetically interacting with *SCH9* in our SGA screening. (XLSX)

S2 Table Gene ontology analysis of genes genetically interacting with *SCH9*. (XLSX)

S3 Table Detailed statistical analysis of CLS and pHv measurements displayed in Fig 5. (XLSX)

S4 Table Yeast strains used in this study. (DOCX)

S5 Table Plasmids used in this study. (DOCX)
