# Supplementary figures and images for: The yeast protein kinase Sch9 adjusts V-ATPase assembly/disassembly to control pH homeostasis and longevity in response to glucose availability
Source: PLoS Genet. 2017 Jun 12;13(6):e1006835. doi: 10.1371/journal.pgen.1006835 (PMC5484544; doi:10.1371/journal.pgen.1006835)

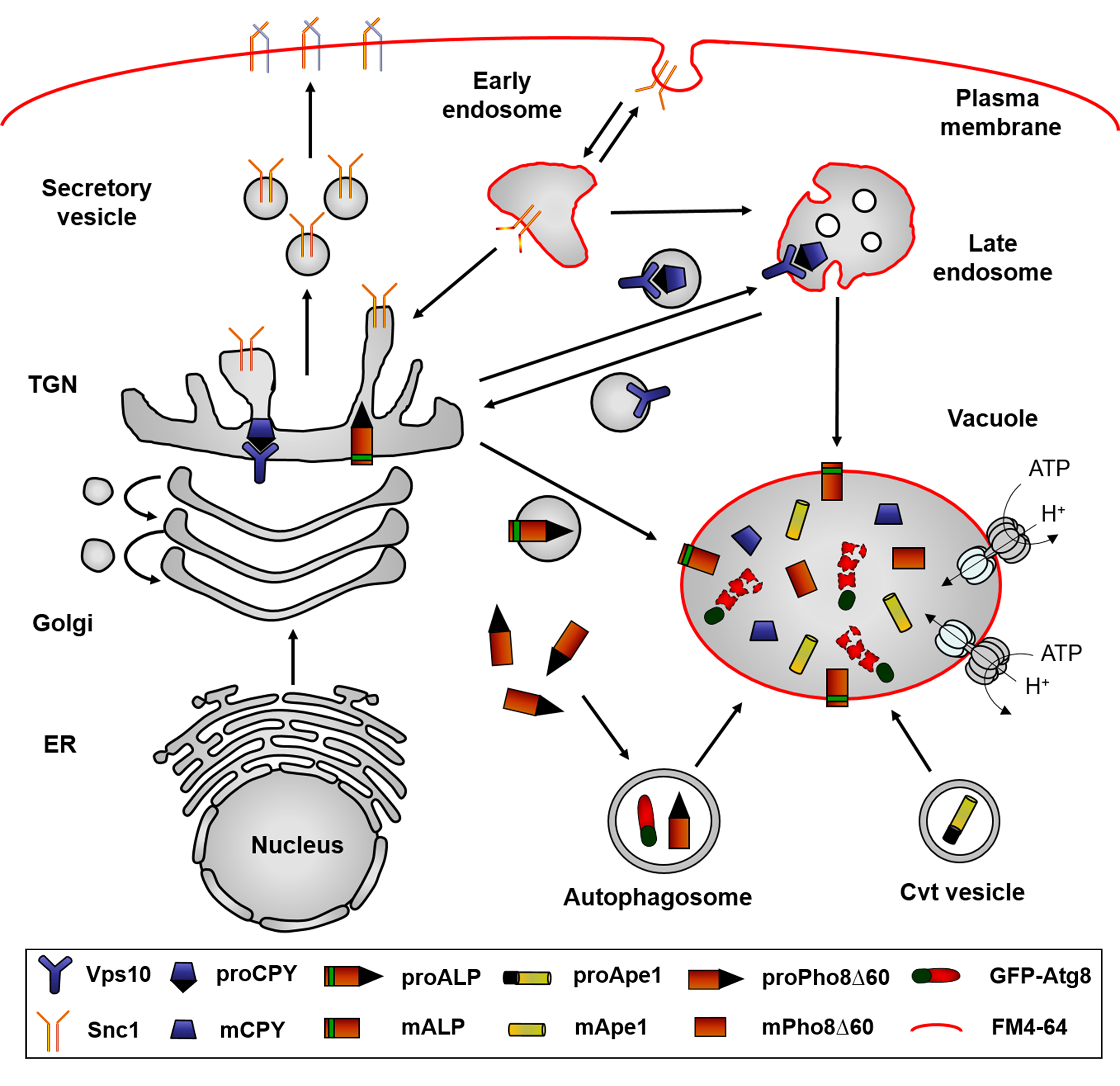

Supplement: S1 Fig — Proteins destined for the vacuole and plasma membrane are sorted in vesicles at the level of the trans-Golgi network (TGN). Upon arrival in the vacuole, proteins are either degraded or cleaved to its active form. The soluble protease proCPY binds the Vps10 receptor in the TGN and this receptor-ligand complex travels to the late endosome. In this compartment, proCPY dissociates from Vps10 and continues its journey to the vacuolar lumen, whereas Vps10p returns to the Golgi via retrograde transport that requires the retromer complex. A direct pathway from the TGN to the vacuole is taken by the vacuolar membrane protein ALP, encoded by PHO8. The v-SNARE protein Snc1, which is involved in the fusion of secretory vesicles with the cell surface, takes a route from the TGN directly to the plasma membrane. After vesicle fusion, Snc1 is recycled from the cell surface to the TGN via the early endosome. Endocytosis sorts proteins from the plasma membrane to the vacuole. The lipophilic dye FM4-64 fluoresces strongly after binding to the plasma membrane and is endocytosed to the vacuole by passing through both early and late endosomes. Cytosolic proteins can be transported to the vacuole by either a selective or a non-selective mechanism. The vacuolar aminopeptidase Ape1 is transported into the vacuolar lumen via the selective cytoplasm-to-vacuole-targeting (Cvt) pathway, while GFP-Atg8 and Pho8Δ60 are non-specifically transported via autophagy. Both processes use double-membrane vesicles to sequester their cargo. Related to Fig 2. (TIF) [file pgen.1006835.s001.tif]

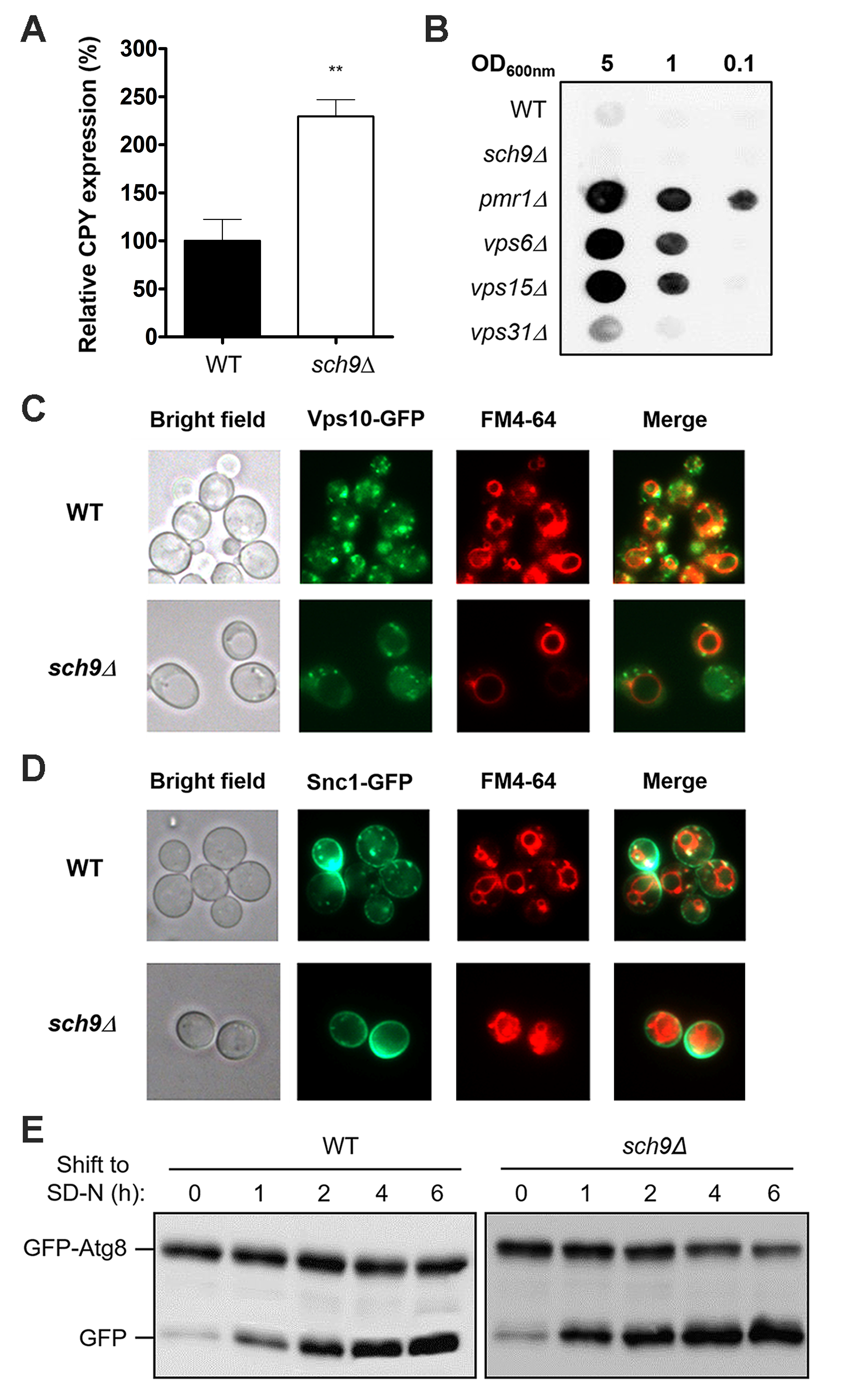

Supplement: S2 Fig — (A) Sch9 regulates CPY abundance. CPY signals were quantified and normalized for Adh2 levels. Results are expressed relative to the WT strain, which was set at 100%. The mean values ± SD from three independent cultures are shown (unpaired t-test). (B) WT and sch9Δ strains do not secrete the soluble hydrolase CPY. Stationary phase cells were spotted and overlaid with a nitrocellulose membrane. After 24h, the membrane was removed, washed and subjected to immunoblotting. (C, D) Sch9 does not influence steady state localization of Vps10-GFP (C) or GFP-Snc1 (D). FM4-64 served as marker for the vacuolar membrane. (E) Delivery and lysis of autophagic bodies is not impaired in the sch9Δ mutant. WT and sch9Δ strains expressing GFP-Atg8 were grown to exponential phase and shifted to nitrogen starvation medium. At the indicated time points, samples were taken. TCA-extracted proteins were analyzed by immunoblotting using anti-GFP antibody. Related to Fig 2. (TIF) [file pgen.1006835.s002.tif]

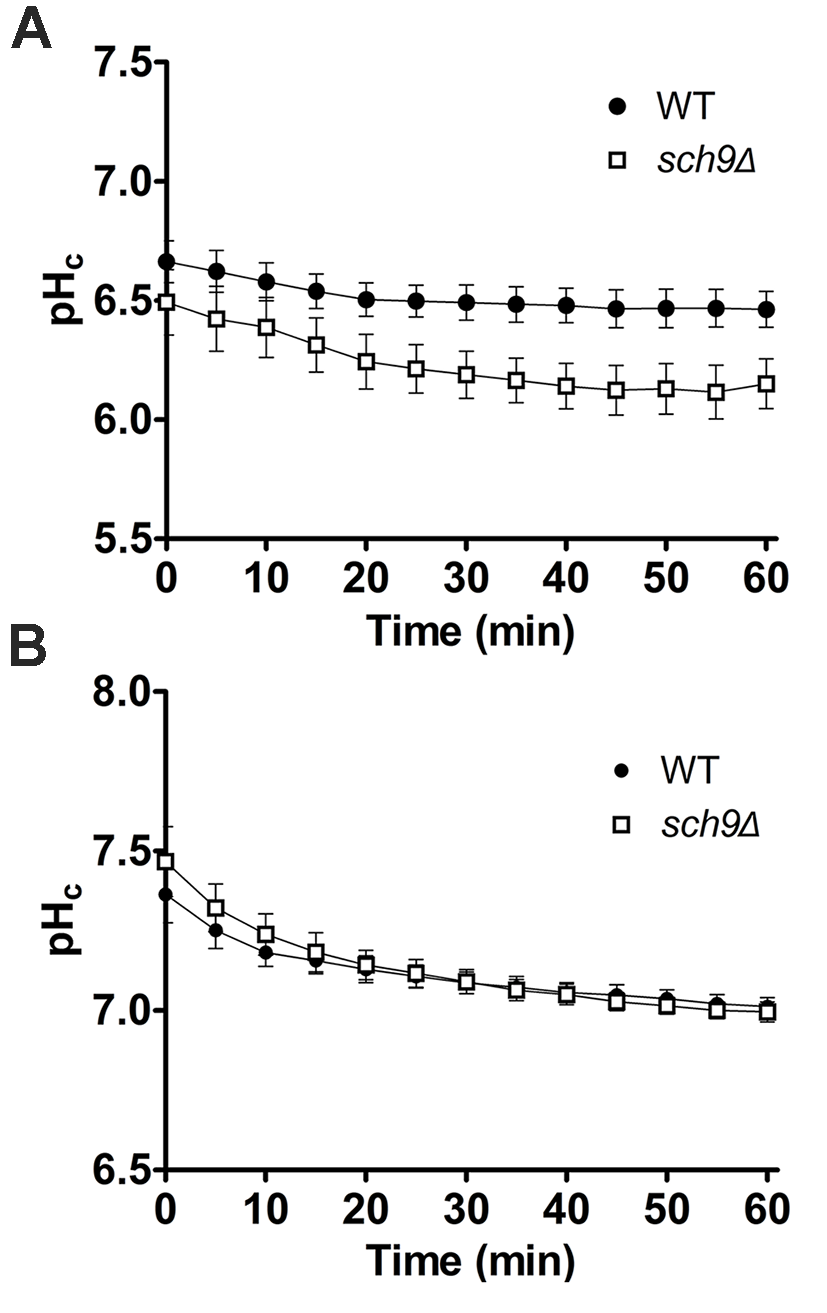

Supplement: S3 Fig — Sch9 affects glucose starvation-induced acidification of the cytosol (A), while nitrogen starvation in general does not impact on pHc homeostasis (B). Cells expressing the pH-sensitive GFP-derivative pHluorin were grown to exponential phase in loflo medium buffered at pH 5, washed twice with starvation medium and transferred to a 96-well microtiter plate. Fluorescence was measured every 5 min for 1h at 30°C in glucose (A) or nitrogen (B) starvation medium. Related to Fig 3. (TIF) [file pgen.1006835.s003.tif]

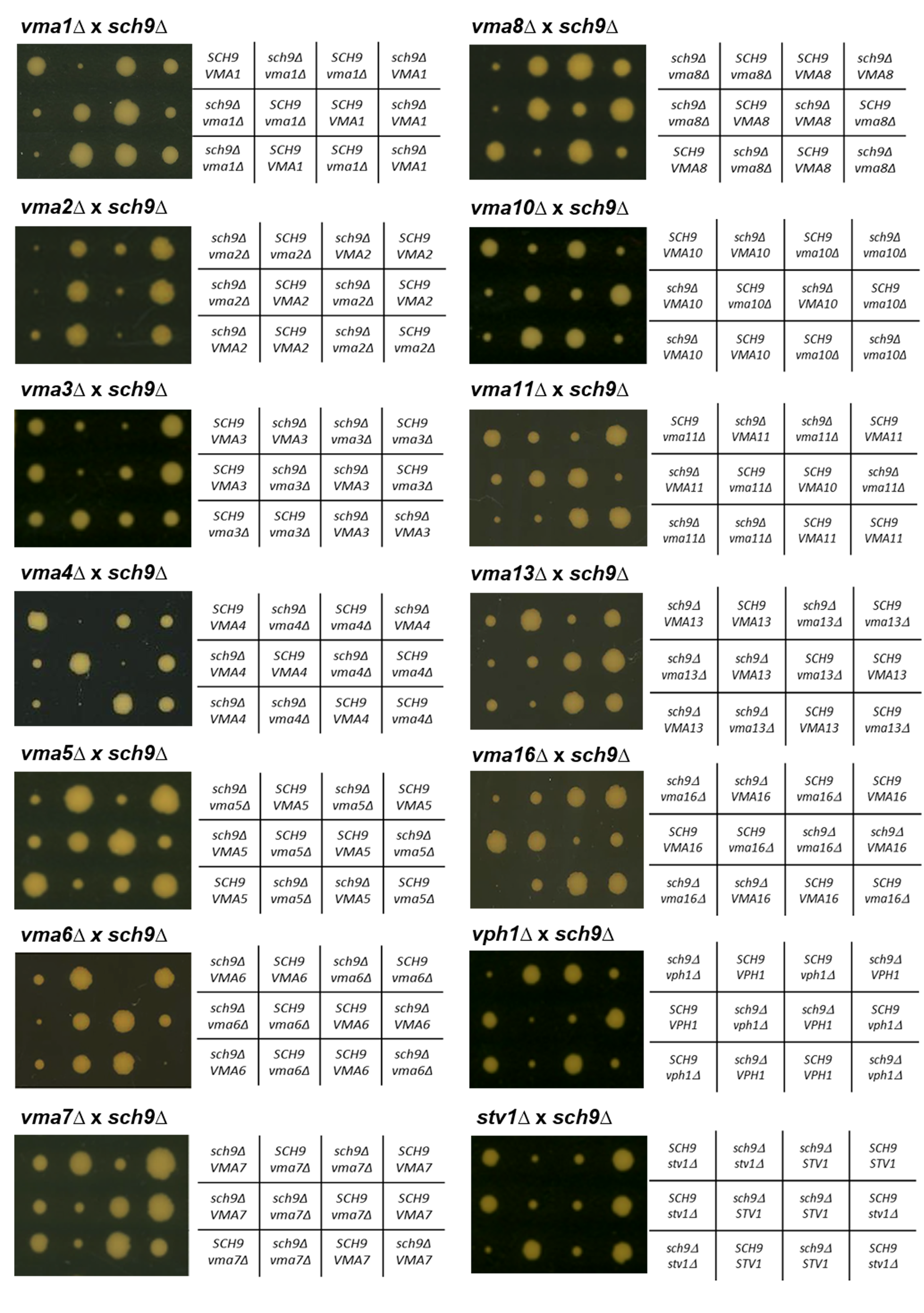

Supplement: S4 Fig — Diploids, generated by crossing the sch9Δ strain (JW 04 039) with the respective single BY4741 deletion strains (EUROSCARF Yeast Knockout Collection), were sporulated and tetrads dissected on YPD (in horizontal rows). Genotypes were determined and are indicated on the right. Related to Fig 4. (TIF) [file pgen.1006835.s004.tif]

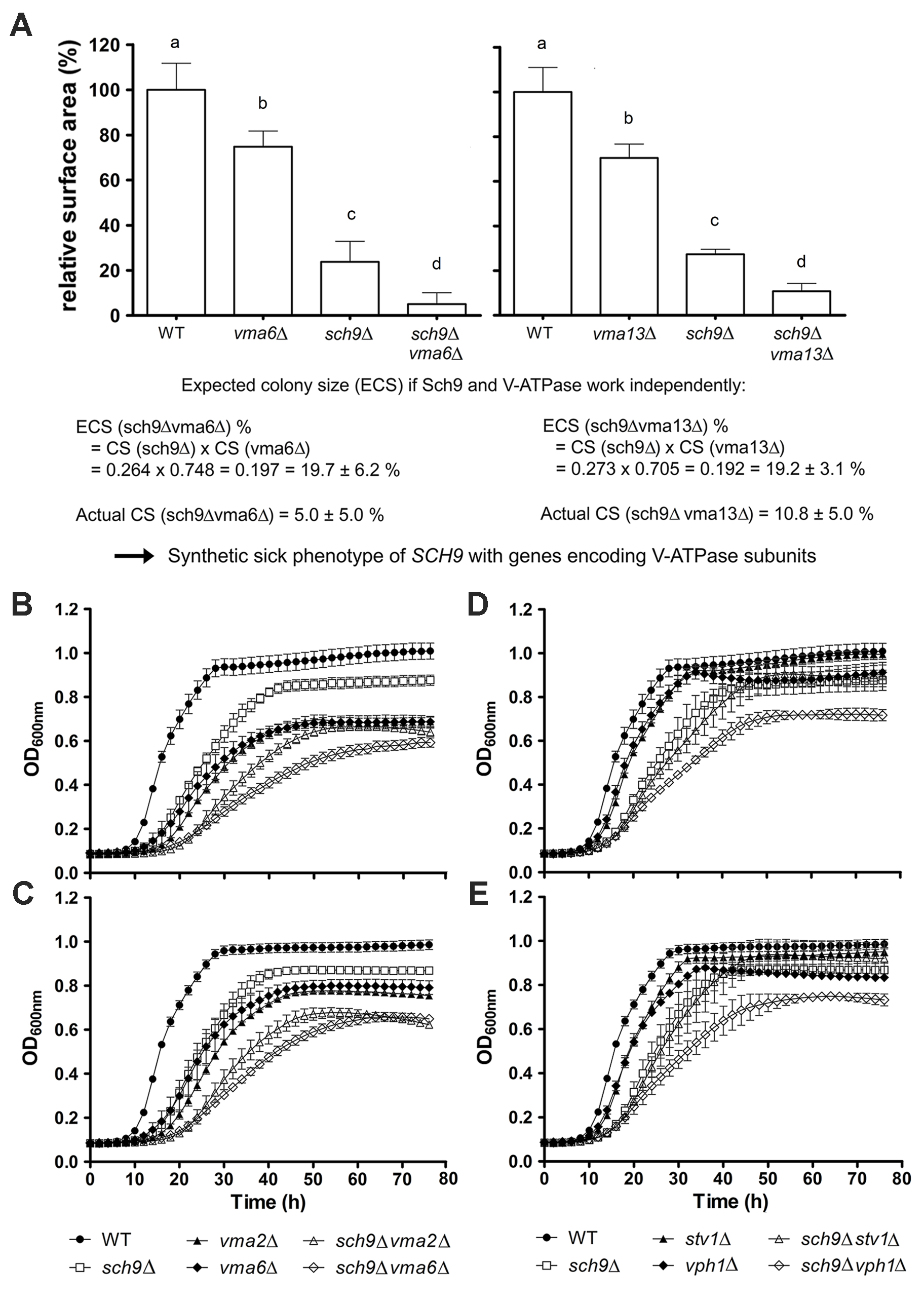

Supplement: S5 Fig — (A) Examples of quantitative analysis of synthetic sick phenotype. Colony sizes (CS) were calculated with ImageJ, using a minimum of 7 independent colonies for each genotype. CS of single and double deletion strains were normalized relative to WT and the expected colony sizes (ECS) for the double deletion mutants were calculated. Results are shown as mean values ± SD. Letters indicate groups of strains with significant difference in colony size (p < 0.01, one-way ANOVA). (B-E) Growth profiles of the indicated single and double deletion mutants. Growth analysis of vma2Δ and vma6Δ (B-C), or the semi-redundant V0 subunits stv1Δ and vph1Δ (D-E) of the V-ATPase reveals a growth defect for strains in which a deletion of SCH9 is combined with a fully dysfunctional V-ATPase. Cultures were pregrown to stationary phase and diluted at the same density in fully supplemented synthetic medium unbuffered (B, D) or buffered to pH 5 (C, E). The mean values ± SD of four independent colonies for each strain are shown. Related to Fig 4. (TIF) [file pgen.1006835.s005.tif]

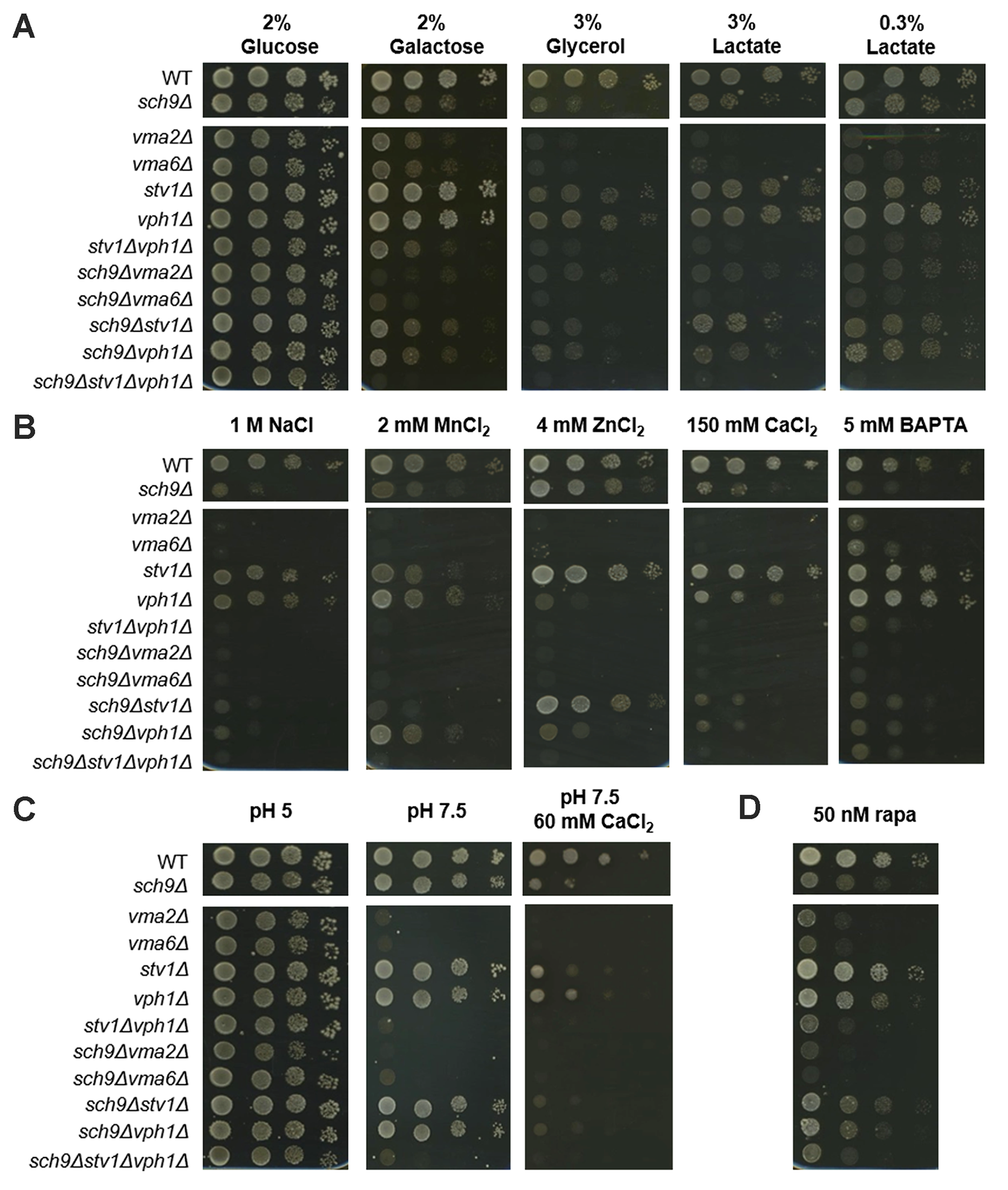

Supplement: S6 Fig — The sch9Δ strain produces a partial vma- phenotype. Stationary phase cells were diluted to an OD600nm of 1 in growth medium, 10-fold serial diluted and spotted on media known to impair growth of either the sch9Δ strain or V-ATPase deficient mutants. (A) Carbon source dependent growth. Various carbon sources were added at the indicated concentration to YP medium. (B) Salt, metal and calcium dependent growth. YPD medium was supplemented with the indicated amount of salt, metal, calcium or calcium chelator. (C) pH sensitive growth. The pH of YPD medium was buffered to pH 5 with 50 mM MES or 7.5 with 100 mM MOPS. (D) Drug sensitivity. Rapamycin was added to YPD medium at a final concentration of 50 nM. Related to Table 1. (TIF) [file pgen.1006835.s006.tif]

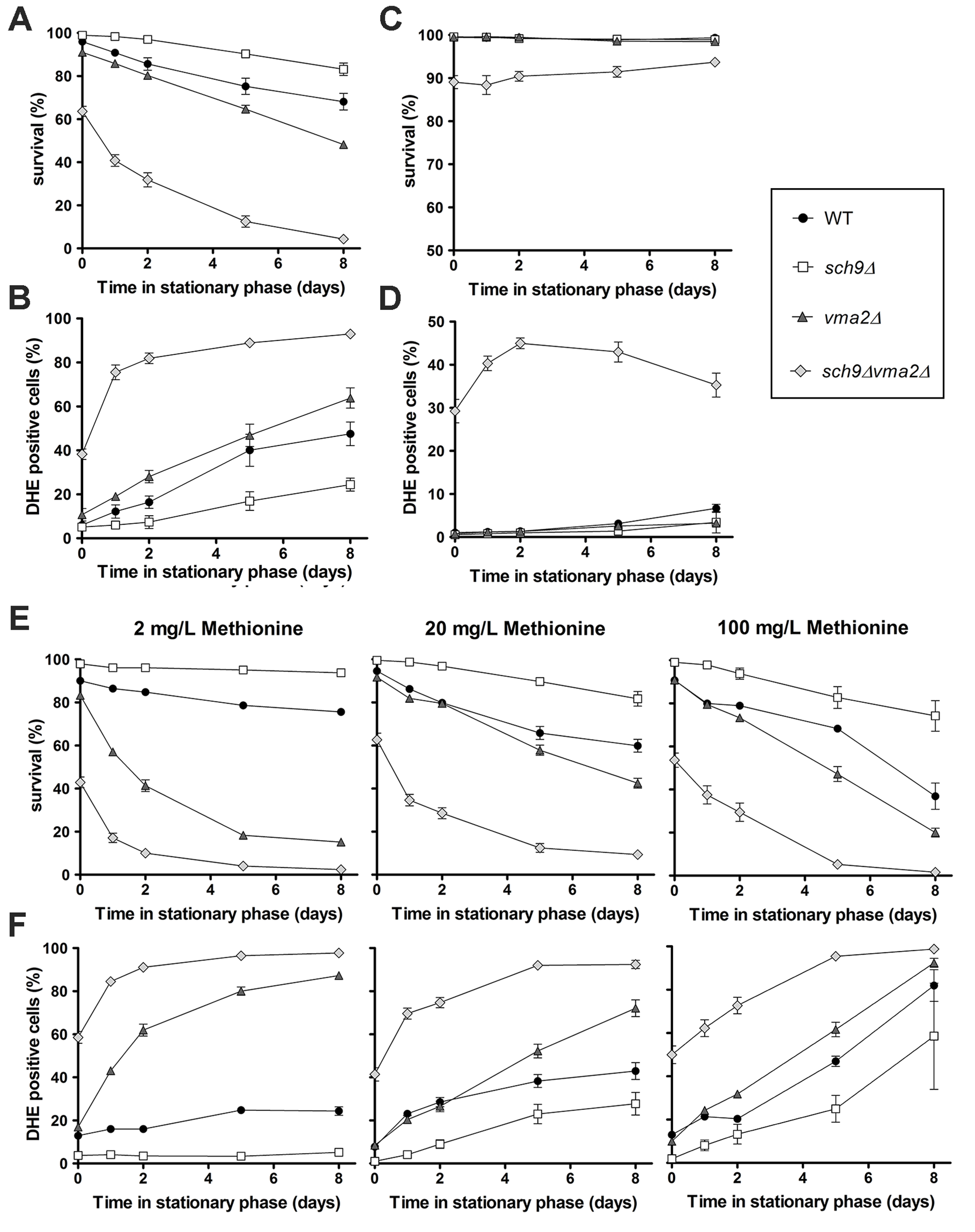

Supplement: S7 Fig — (A) Chronological ageing and (B) ROS accumulation over time of strains grown in non-buffered fully supplemented medium. (C) Cell survival and (D) ROS levels of strains grown in fully supplemented medium buffered at pH 5.5 with 100 mM MES. (E) Cell survival and (F) ROS levels of strains grown in medium containing the indicated concentration of methionine. For all experiments, stationary phase cells were inoculated in fresh medium at OD600nm 0.1, grown for 48h (day 0), and stained with SYTOXgreen and DHE at the indicated time points. Results depicted are mean values ± SD. Related to Fig 5. (TIF) [file pgen.1006835.s007.tif]

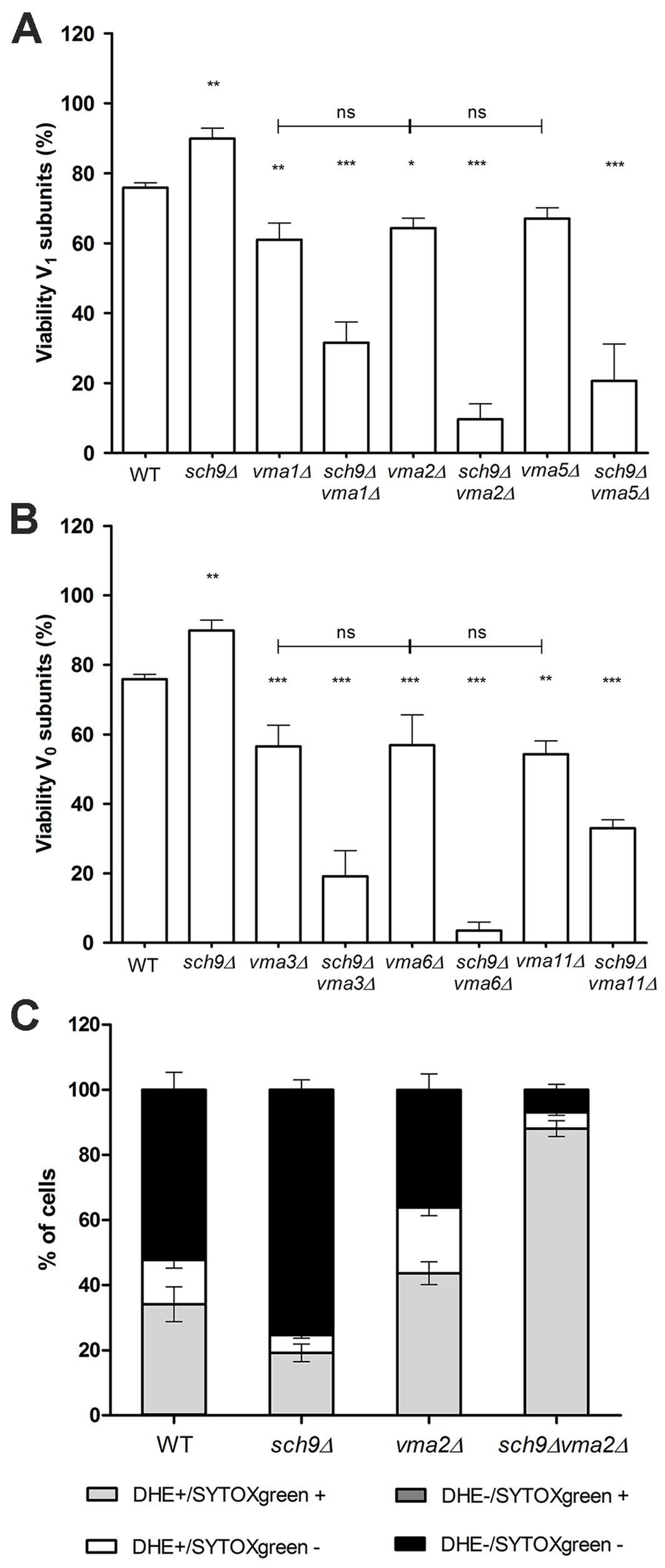

Supplement: S8 Fig — Chronological ageing of V1 (A) and V0 subunits (B) of the V-ATPase in combination with deletion of SCH9 was assessed by staining stationary phase cells with propidium iodide (PI). For each strain, the amount of PI positive (death cells) and PI negative cells (viable cells) was determined. (C) Stationary phase cells were co-stained with SYTOXgreen and DHE to detect loss of membrane integrity and superoxide accumulation, respectively. Results are shown as the average of at least three independent clones for each strain, error bars represent SD. Asterisks indicate a statistical significance compared to the WT strain (one-way ANOVA analysis). Related to Fig 5. (TIF) [file pgen.1006835.s008.tif]

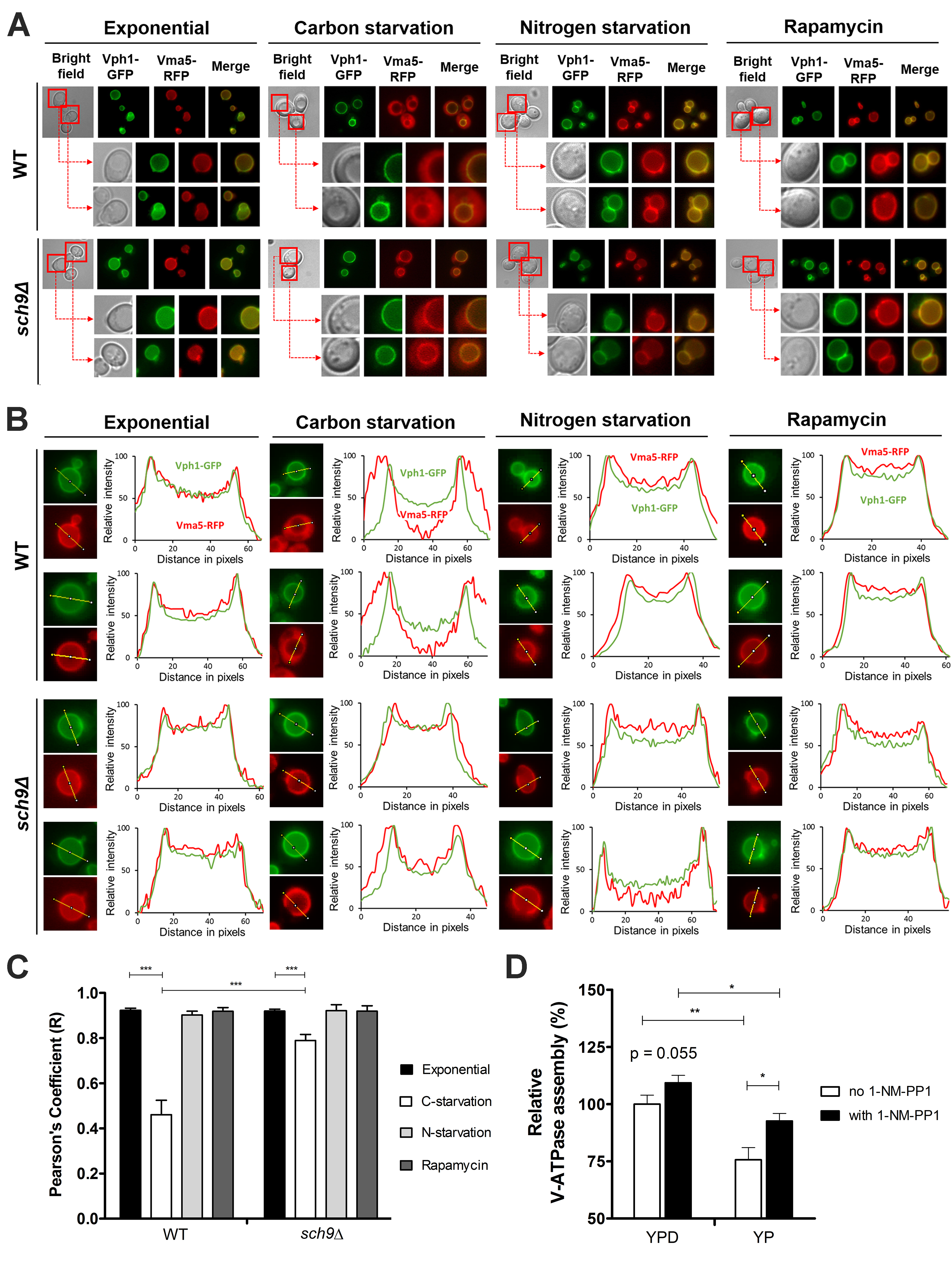

Supplement: S9 Fig — (A) WT and sch9Δ cells co-expressing Vma5-RFP and Vph1-GFP were grown as in Fig 6A and their intracellular localization was analyzed by fluorescence microscopy. In contrast to WT, in sch9Δ cells a significant portion of Vma5-RFP was still found at the vacuolar membrane during glucose starvation. (B) Fluorescence intensity profile plots. Combined fluorescence intensity profile plots of Vma5-RFP (red) and Vph1-GFP (green) measured along the line displayed in the panels on the left for WT and sch9Δ cells. The x-axis depicts the distance along the line in pixels, while the y-axis indicates the relative RFP or GFP signal intensities. (C) The Pearson’s coefficient was calculated using the ImageJ plugin JACoP. Results depicted are mean values ± 95% CI. (one-way ANOVA analysis). (D) V-ATPase assembly and disassembly levels in the sch9as strain. Cultures were grown in YPD medium with or without 300 nm 1-NM-PP1 for 6 hours, after which they were starved for glucose (30 min) in the absence or presence of the inhibitor. V-ATPase assembly levels were calculated, normalized relative to cells grown on YPD medium without inhibitor and are shown as mean values ± SEM. Statistical significance was tested by a two-way ANOVA analysis (Holm-Sidak's multiple comparisons test). Related to Figs 6 and 7. (TIF) [file pgen.1006835.s009.tif]

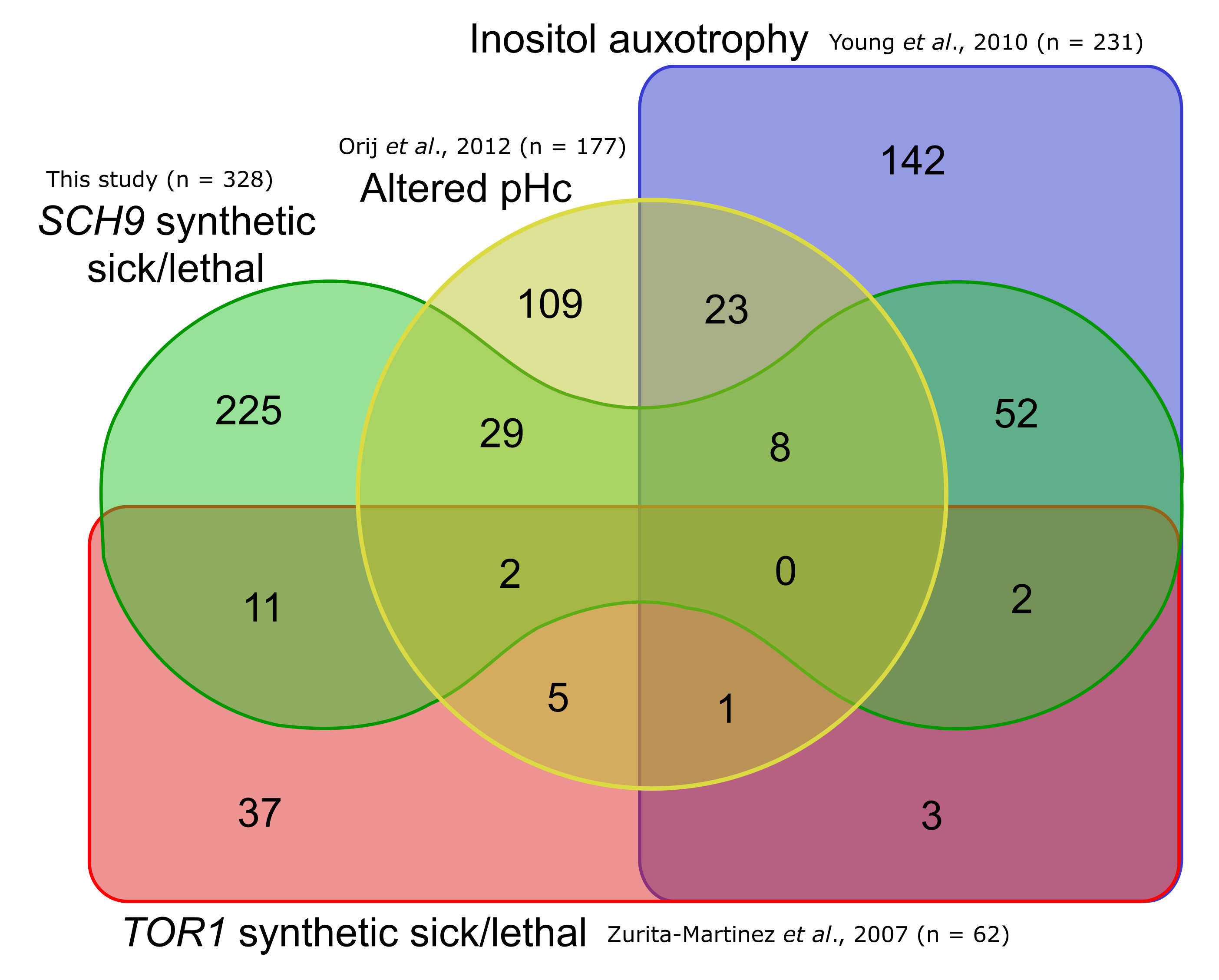

Supplement: S10 Fig — A significant overlap of the indicated screenings with our SGA screening implicates Sch9 as central player connecting inositol and lipid metabolism with nutrient availability, pHc, V-ATPase activity and growth. (TIF) [file pgen.1006835.s010.tif]
