# Supplementary material for: The yeast protein kinase Sch9 adjusts V-ATPase assembly/disassembly to control pH homeostasis and longevity in response to glucose availability
Source: PLoS Genet. 2017 Jun 12;13(6):e1006835. doi: 10.1371/journal.pgen.1006835 (PMC5484544; doi:10.1371/journal.pgen.1006835)
Supplement: S4 Table — (DOCX) [file pgen.1006835.s014.docx]

**Supplemental Table S4: Yeast strains used in this study**

| **Name** | **Relevant genotype** | **Source** |
| --- | --- | --- |
| BY4741 (WT) | MATa *his3Δ1 leu2Δ0 met15Δ0 ura3Δ0* | Openbiosystems |
| JW 04 038 | MATa *his3Δ1 leu2Δ0 met15Δ0 ura3Δ0 sch9∆::NATMX4* | This study |
| JW 04 039 | MATα *his3Δ1 leu2Δ0 met15Δ0 ura3Δ0 sch9∆::NATMX4* | This study |
| JW 04 439 (RD67) | BY4741 with *VMA2-GFP::HIS3* | (Dechant *et al*., 2010) |
| JW 04 441 | JW 04 038 with *VMA2-GFP::HIS3* | This study |
| JW 04 439 (RD157) | BY4741 with *VMA5-RFP::KANMX4 VPH1-GFP::HIS3* | (Dechant *et al*., 2010) |
| JW 04 442 | BY4741 with *VMA5-RFP::KANMX4* | This study |
| JW 04 446 | JW 04 038 with *VMA5-RFP::KANMX4* | This study |
| JW 04 554 | BY4741 with *VPS10-GFP::HIS3* | This study |
| JW 04 556 | JW 04 038 with *VPS10-GFP::HIS3* | This study |
| JW 04 559 | BY4741 with *pho8∆::hphNT1* | This study |
| JW 04 560 | JW 04 038 with *pho8∆::hphNT1* | This study |
| JW 03 999 | BY4741 with *SCH9^T492G^* (*sch9as*) | (Jorgensen *et al*., 2004) |
| JW 10 413 | BY4741 with *prc1∆::KANMX4* | Y.K.O collection |
| JW 14 457 | BY4741 with *pho8∆::KANMX4* | Y.K.O collection |
| JW 10 809 | BY4741 with *pep4∆::KANMX4* | Y.K.O collection |
| JW 01 910 | BY4741 with *vma1∆::KANMX4* | Y.K.O collection |
| JW 14 326 | BY4741 with *vma2∆::KANMX4* | Y.K.O collection |
| JW 11 096 | BY4741 with *vma3∆::KANMX4* | Y.K.O collection |
| JW 10 605 | BY4741 with *vma4∆::KANMX4* | Y.K.O collection |
| JW 11 615 | BY4741 with *vma5∆::KANMX4* | Y.K.O collection |
| JW 13 406 | BY4741 with *vma6∆::KANMX4* | Y.K.O collection |
| JW 12 231 | BY4741 with *vma7∆::KANMX4* | Y.K.O collection |
| JW 11 115 | BY4741 with *vma8∆::KANMX4* | Y.K.O collection |
| JW 12 925 | BY4741 with *vma10∆::KANMX4* | Y.K.O collection |
| JW 10 745 | BY4741 with *vma11∆::KANMX4* | Y.K.O collection |
| JW 13 199 | BY4741 with *vma13∆::KANMX4* | Y.K.O collection |
| JW 13 324 | BY4741 with *vma16∆::KANMX4* | Y.K.O collection |
| JW 14 623 | BY4741 with *stv1∆::KANMX4* | Y.K.O collection |
| JW 14 228 | BY4741 with *vph1∆::KANMX4* | Y.K.O collection |
| JW 04 928 | BY4741 with *vph1∆::KANMX4 stv1∆::KANMX4* | This study |
| JW 04 474 | BY4741 with *sch9∆::NATMX4 vma1∆::KANMX4* | This study |
| JW 04 454 | BY4741 with *sch9∆::NATMX4 vma2∆::KANMX4* | This study |
| JW 04 456 | BY4741 with *sch9∆::NATMX4 vma3∆::KANMX4* | This study |
| JW 04 562 | BY4741 with *sch9∆::NATMX4 vma4∆::KANMX4* | This study |
| JW 04 458 | BY4741 with *sch9∆::NATMX4 vma5∆::KANMX4* | This study |
| JW 04 377 | BY4741 with *sch9∆::NATMX4 vma6∆::KANMX4* | This study |
| JW 04 462 | BY4741 with *sch9∆::NATMX4 vma7∆::KANMX4* | This study |
| JW 04 464 | BY4741 with *sch9∆::NATMX4 vma8∆::KANMX4* | This study |
| JW 04 466 | BY4741 with *sch9∆::NATMX4 vma10∆::KANMX4* | This study |
| JW 04 383 | BY4741 with *sch9∆::NATMX4 vma11∆::KANMX4* | This study |
| JW 04 564 | BY4741 with *sch9∆::NATMX4 vma13∆::KANMX4* | This study |
| JW 04 387 | BY4741 with *sch9∆::NATMX4 vma16∆::KANMX4* | This study |
| JW 05 039 | BY4741 with *sch9∆::NATMX4 stv1∆::KANMX4* | This study |
| JW 05 035 | BY4741 with *sch9∆::NATMX4 vph1∆::KANMX4* | This study |
| JW 04 948 | BY4741 with *sch9∆::NATMX4 vph1∆::KANMX4 stv1∆::KANMX4* | This study |
