# Supplementary material for: The yeast protein kinase Sch9 adjusts V-ATPase assembly/disassembly to control pH homeostasis and longevity in response to glucose availability
Source: PLoS Genet. 2017 Jun 12;13(6):e1006835. doi: 10.1371/journal.pgen.1006835 (PMC5484544; doi:10.1371/journal.pgen.1006835)
Supplement: S5 Table — (DOCX) [file pgen.1006835.s015.docx]

**Supplemental Table S5: Plasmids used in this study**

| **Name** | **Backbone** | **Marker** | **Insert** | **Source** |
| --- | --- | --- | --- | --- |
| CPY-GFP | pRS313 | *HIS3* | *pTEF1pr-CPY-GFP* | Y. Kimata |
| pJLU40 | pRS315 | *LEU2* | *pVPH1pr-VPH1-GFP* | C. Piper |
| pJU793 | pRS416 | *URA3* | *pSCH9pr-GFP-SCH9* | R. Loewith |
| pJU677 | pRS416 | *URA3* | *pSCH9pr -6HA-SCH9^WT^* | R. Loewith |
| pJU790 | pRS416 | *URA3* | *pSCH9pr- 6HA-SCH9^5A^* | R. Loewith |
| pJU855 | pRS416 | *URA3* | *pSCH9pr- 6HA-SCH9^2D3E^* | R. Loewith |
| FBp1004 | pRS415 | *LEU2* | *pSCH9pr-GFP-SCH9* | This study |
| pGF242 | pRS316 | *URA3* | *pVPH1pr-mCherry-PHO8* | T. H. Stevens |
| GFP-ALP | pRS426 | *URA3* | *pCPY1pr-GFP-PHO8* | S. Emr |
| GFP-Aut7 | pRS316 | *URA3* | *pATG8pr-GFP-ATG8* | D. Klionsky |
| pCB506 | Ycp | *URA3* | *pSnc1pr-GFP-SNC1* | V. Bankaitis |
| cytPho8Δ60 | pYX242 | *LEU2* | *pTPIpr-cytPHO8Δ60* | P. Ludovico |
| pACT1-pHluorin | pYES2 | *URA3* | *pACT1pr-pHluorin* | G. Smits |
| pHLUM | pRS313 | *HIS3* | *MET15, LEU2, URA3* | M. Ralser |
| FBp910 | pRS313 | *HIS3* | *MET15, LEU2* | This study |
